# Extended Hierarchical Fuzzy Interpreted Petri Net

**DOI:** 10.3390/s21248433

**Published:** 2021-12-17

**Authors:** Michał Markiewicz, Lesław Gniewek, Dawid Warchoł

**Affiliations:** Department of Computer and Control Engineering, Faculty of Electrical and Computer Engineering, Rzeszów University of Technology, W. Pola 2, 35-959 Rzeszów, Poland; mmarkiewicz@prz.edu.pl (M.M.); lgniewek@prz.edu.pl (L.G.)

**Keywords:** Petri nets, fuzzy nets, interpreted nets, hierarchical nets, computer simulation, control systems

## Abstract

Petri nets (PNs) have many advantages such as graphical representation, formal description, and the possibility of sequential and concurrent control. An important aspect of using PNs is hierarchical modeling, which may be provided in different ways. In this paper, a new concept and definition of the hierarchical structure for Fuzzy Interpreted Petri Net (FIPN) are proposed. The concept of macroplace with several input, output, and input-output places is introduced to the net. The functionality of the macroplace instances and the hierarchy graph are also proposed. They are implemented in a computer simulator called HFIPN-SML. In this study, FIPN is employed since it allows the use of analogue sensors directly for process control. Better visualization and more precise control are among advantages of the introduced approach.

## 1. Introduction

Petri nets (PNs) are increasingly used in the development of information systems whose high complexity requires a new approach to design and implementation. Therefore, new concepts of PN description, analysis, and presentation have appeared, among which an important place takes introducing the hierarchy (modularization) to the net structure. It allows for the presentation of the developed systems in different levels of abstraction and facilitates the determination of the properties of nets modeling these systems. There is, however, no unified approach to using the hierarchy in PNs, and the main research directions are described in the following subsections.

### 1.1. Refinement of Places and Transitions

One of the first works related to the hierarchy in PNs was presented by Valette [1], who proposed a concept of macrotransition with a single input and output. The author defined a concept of a well-formed block and examined the properties of the hierarchical net, such as boundedness, safeness, and liveness. Suzuki and Murata [2] continued Valette’s research by extending the concept of the well-formed block to a k-well-behaved block. Vogler [3] proposed an extension of the concepts described in [1,2] by replacing a transition having several inputs and outputs with a subnet taking into account certain constraints of the structure. Brauer et al. [4] described the refinement of transitions for a subnet with one initial and one final transition as well as a method for creating macroplaces.

Bernardinello and De Cindio [5] presented and compared different approaches to the refinements of place and transition. The majority of these concepts use the composition of synchronized subnets with a state machine structure. Van Der Aalst [6] described a method to validate the workflow using a hierarchical net, which preserves properties such as safeness, liveness, boundedness, free-choice, and well-structuredness. The net was used to model the flow control of different aspects of business processes. Huang et al. [7] described a method for refinement providing nineteen system properties. In the literature, a refinement of actions and operations is often considered [4,8,9,10,11,12]. Such an approach also allows representing a system hierarchically and its analysis in different levels of abstraction.

### 1.2. Reduction

Another approach to the modeling of the hierarchy in PNs is a reduction technique. It allows determining static and dynamic properties of a net based on its smaller counterpart to which it has been transformed. The first research on this topic appeared in the seventies [13,14,15,16]. These works have been generalized by Berthelot [17,18], who proposed transformations providing properties such as liveness, reachable marking, proper termination, home state, deadlock freeness, unavoidable states, and abstraction. The following transformations can be distinguished: transformation of places, fusion of transitions, S-decomposition, and T-decomposition, which are based mainly on the static properties. Lee et al. [19,20] proposed a hierarchical reduction method that uses a decomposition. The method, based on a net structure, allows preserving properties such as liveness, boundedness, and proper termination.

Desel, Best and Esparza [21,22,23,24] described the reduction of free choice nets. In [21,22,23], they proposed the use of four types of reduction: P-reduction, T-reduction, F-reduction, and A-reduction. Desel and Esparza [24] summarized the results related to free choice nets and their properties. Jiao et al. [25,26] continued the research of Desel, Best, and Esparza and studied the properties of asymmetric free choice net. In the literature, the application of reduction rules for time PNs were also described [27,28,29].

### 1.3. Formal Description of the Hierarchical PNs

Introducing the hierarchy in PNs by a formal description and a net definition allows imposing certain initial constraints (such as the number of net inputs and outputs) and creating subnets in any way and with any properties.

One of the first works presenting the definition of a hierarchical net is the study of Fehling [30], in which a combination of a hierarchical PN with a morphism is presented. One of the most complete concepts is, however, the Colored Petri Net (CPN) described by Jensen [31]. The author proposed a definition, according to which a net can consist of separate subnets (called pages) that are related to transitions. Instances of places, transitions, arcs, and subnets were also described. Additionally, it is possible to define the so-called fusion of places. The relations between the subnets can be visualized with a hierarchy graph.

Holvoet and Verbaeten [32] presented an alternative definition of a hierarchical net. A recursion is used in the formal description. He [33] introduced the formal definition of hierarchical PN for modeling large, complex, and parallel distributed systems which uses the concept of macrotransitions and macroplaces. In the proposed solution, the hierarchical net has a tree structure. Andrzejewski [34] presented a formal specification of a reactive system based on a hierarchical interpreted time PN, which has a state memory in different levels of the hierarchy. In turn, Pan and Sun [35] proposed a hierarchical fuzzy PN, which can be used for decision systems modeling. In the net, both the abstract places and transitions (macroplaces and macrotransitions) are defined.

### 1.4. Hierarchical Nets in Industrial Standards

The formalism of PNs along with their hierarchy structure has found its application in programming standards of industrial controllers. The use of PNs to write programs for PLC controllers is described in the international standard IEC 60848:2013 [36], that proposes a Grafcet language. Based on Grafcet, the Sequential Function Chart (SFC) is defined [37] as one of the five programming languages for PLC controllers. The theoretical basis of Grafcet are described by David and Alla [38].

In the Grafcet standard, it is possible to use several functionalities based on the hierarchy. The first of them is the concept of the macroplace called macrostep. It assumes that a token, which is moved to a macroplace node, is automatically moved to the input step of a subnet assigned to this macrostep. Another concept is the so-called enclosure, in which a subnet is assigned to one enclosure step. If a token moves to the enclosure step, the initial steps of the subnet are activated, and when the token leaves the enclosure step, all subnet steps are deactivated. A different functionality is forcing subnet states: pausing and resuming a subnet work as well as activating/deactivating of the chosen steps.

In the IEC 61131-3 standard, the hierarchical structure is not directly defined for the SFC language. However, the way in which the actions assigned to steps are defined indirectly introduces the hierarchical nesting. They can be implemented using SFC and other languages of this standard.

### 1.5. Methodology of Dealing with Complex Systems

Modular PNs can be used for the creation of methodologies that describe dealing with complex systems, e.g., manufacturing systems. This approach is usually given in the form of a general verbal description supplemented with examples and formal descriptions.

Silva and Valette [39] proposed the modeling of Flexible Manufacturing Systems (FMS) using hierarchical PNs. Stepwise place/transition refinements and modular composition were applied. Valavanis [40] described a methodology of FMS modeling using an extended PN. In the approach, a decomposition (top-down) and composition (bottom-up) are used. Zhou et al. [41] proposed a hybrid methodology that ensures the appropriate properties of modules, such as boundedness, liveness, and reversibility. In the described concept, the decomposition and composition are employed for the determination of unshared and shared resources between modules.

Der Jeng and DiCesare [42] described the use of PNs in the modeling of shared-resource automated manufacturing systems. The merging of the modules is performed through transitions shared by different subnets. The methodology allows the preservation of liveness and boundedness. Zhou [43] showed a practical example of modular modeling of a semiconductor manufacturing system using time PN. A reduction technique is used to facilitate the analysis of properties of the entire system.

### 1.6. Recent Studies

Recent studies indicate that the hierarchy/modularization in PNs can be very useful. One of the subjects of research is decomposition. Ye et al. [44] showed the method for the creation of the decentralized control system using PNs. In the proposed solution, the subnets have a state-machine structure. Wisniewski et al. proposed a new algorithm of decomposition for edge systems modeled using Petri nets [45]. Formal proof of the correctness of the algorithm and comparison to a similar method were also given. For the modeling of practical problems, hierarchical Colored Petri Nets (HCPN) are widely used. The application of HCPN for the implementation of a traffic signal control model was proposed by An et al. [46]. Sicchar et al. [47] presented the application of HCPN in the control system for load-balance procedure in low voltage grid. Gozhyj et al. [48] presented the use of HCPN to model the interaction between web services using CPN tools.

Fuzzy hierarchical PNs and formal definitions of hierarchy are also considered. Li et al. [49] presented a layered fuzzy Petri net with a hierarchical structure for risk assessment. The fault diagnosis approach using time hierarchical fuzzy Petri net was proposed by Yuan et al. [50]. The combination of fuzzy and colored PNs for runtime veryficaton of pacemaker was presented by Majma et al. [51]. Padberg [52] proposed a formal definition of hierarchical reconfigurable PN and its conversion to a flat net. Different models and problems related to hierarchical PNs can also be found in [53,54,55,56].

### 1.7. Paper Scope and Organization

As can be seen from the previous subsections, the hierarchy in PNs can be realized in different ways. The boundary between the discussed research areas is ambiguous because they often overlap and can complement each other. For example, an important methodology for dealing with large systems, such as FMS, can be the reduction technique [43,57,58].

In this paper, a new concept and a formal description of hierarchical FIPN (HFIPN) is presented. The theoretical basis of FIPN are described in [59], where the structure, behavior, and algebraic representation of this net are shown. In [60], a coverabality graph for FIPN was proposed, which can be used in the study of net properties and as a diagnostic system. The properties of FIPN can also be determined using a reachability graph. Research related to the FIPN implementation is also conducted. The works [61,62] describe the FIPN software model and its implementation for PLC controllers, while a method of code generation for FPGA circuits is presented in [63]. As part of the research, a software tool called HFIPN-SML was developed based on (H)FIPN model.

This paper proposes a new hierarchical structure for FIPN and is a continuation of [64]. The contributions of this study are as follows:

(a) Concept of a macroplace that can have several input, output and input-output places;
(b) Functionality of macroplace instances;
(c) Formal definition of HFIPN;
(d) Concept and a definition of a hierarchy graph displaying a hierarchical structure and allowing a quick access to all subnets in an implementation version;
(e) Formal algebraic representation of HFIPN;
(f) Conversion of HFIPN to its flat version;
(g) Formal way to sum any two subnets.

The paper is organized as follows. First, a comparison of our approach to similar solutions is presented (Section 1.8). Then, the theoretical basis of FIPN is described (Section 2). Next, the new HFIPN concept is presented (Section 3). Then, an exemplary application is shown (Section 4). Finally, a summary of the research and potential future studies are given (Section 5).

### 1.8. Comparison to Similar Solutions

Due to previous research, HFIPN is compared to PNs used in PLC controllers. In Table 1 general characteristics of Signal Interpreted Petri Net (SIPN) [65,66,67,68], Grafcet [36], SFC [37], and other fuzzy PNs [69,70,71] are presented. The first three were chosen since they seem to be the most comprehensive because of the hierarchy structure, software tool support, and automatic executable code generation. The others, like HFIPN, allow the use of analogue signals, but they do not have the advantages shown in Table 1 that facilitate the practical application.

SIPN is a binary net with a formal description. It allows the use of a hierarchy based on a macroplace concept with one input and output place. Software based on SIPN enables the automatic conversion of the graphical net representation to executable code in Instruction List (IL) language and the automatic investigation of the net properties. All functionalities are implemented in one computer tool. Grafcet and SFC are binary nets allowing the use of a hierarchical structure (described in Section 1.4) and automatic executable code generation based on a net graph. Although they are proposed as international standards, there are some issues related to their informal description. There is no unified approach to the formal analysis of their properties and their operation depends on implementation. They can be converted to the finite machine [72,73,74], timed automata [72,75,76,77,78], or formal PNs [79,80,81,82,83].

In comparison to the mentioned solutions, the main advantage of HFIPN is the possibility of using analogue signals for direct control of a process. Conditions assigned to a transition may not be binary (it is explained in the next section). Contrary to Grafcet, SFC, and SIPN, HFIPN also allows resource modeling in the structure of a net since the weight of arcs and the capacity of places can be greater than one. In comparison to SIPN, HFIPN offers more possibilities in the use of a hierarchical structure. However, HFIPN has some gaps that need to be filled as shown in Table 1. As with SIPN, SFC, and Grafcet, HFIPN permits automatic generation of executable code [64], but unlike the others, only for a non-hierarchical net structure. Another issue is the investigation of properties. For HFIPN, like for Grafcet and SFC, there are created methods to investigate properties [60], but they are not integrated with HFIPN-SML. Clearing both gaps is planned for future studies.

To conclude this section, it can be observed that there is no other solution combining PNs and PLC programming to create control systems and offering the possibility of using analogue sensors, software tool support, resource modeling by the structure of a net, and automatic generation of executable code. However, some new functionalities need to be implemented to see HFIPN and its simulator as a complete solution.

## 2. The Formal Basis and the Concept of FIPN

Three definitions describe the formal basis of FIPN. The first presents the net construction.

**Definition** **1.** *The fuzzy interpreted Petri net is the system [59]:*

*FIPN $=(P,t,\Omega ,\Psi ,R,\Delta ,K,W,\Gamma ,\Theta ,M_{0},e)$, where:*

*$P=P^{\prime }\cup P^{\prime \prime }$ is a nonempty finite set of places, where:*

   *$P^{\prime }=\left\{p_{1}^{\prime },p_{2}^{\prime },...,p_{a^{\prime }}^{\prime }\right\}$ is a set of places for processes modeling,*

   *$P^{\prime \prime }=\left\{p_{1}^{\prime \prime },p_{2}^{\prime \prime },...,p_{a^{\prime \prime }}^{\prime \prime }\right\}$ is a set of places for resources modeling;*

*$T=\{t_{1},t_{2},...,t_{b}\}$ is a nonempty finite set of transitions;*

*$\Omega =\{\omega_{1},\omega_{2},...,\omega_{a^{\prime }+a^{\prime \prime }}\}$ is a nonempty finite set of statements;*

*$\Psi =\{\psi_{1},\psi_{2},...,\psi_{b}\}$ is a nonempty finite set of conditions;*

*P, T, Ω, Ψ are disjoint sets;*

*R⊆(P×T)∪(T×P) is the incidence relation that assigns a place to each transition $t_{i}$*

   *(1⩽i⩽b) where there is the place $p^{\prime }\in P^{\prime }$, such that $(p^{\prime },t_{i})\in R$ or $(t_{i},p^{\prime })\in R$;*

*Δ:P→Ω is the function assigning a statement to each place;*

*$K:P^{\prime }\to \left\{1\right\}$ and $P^{\prime \prime }\to ℵ\backslash \left\{1\right\}$ are the functions assigning a capacity to each place, where*

   *ℵ={1,2,...};*

*Γ:T→Ψ is the function assigning a condition to each transition;*

*Θ:T→[0,1] is the function defining the degree to which the condition corresponding to the*

   *each transition is satisfied;*

*W:R→ℵ is the weight function which ∀p∈P and ∀t∈T meets two conditions:*

   *W(p,t)≤K(p),*

   *W(t,p)≤K(p);*

*$M_{0}:P^{\prime }\to \left\{0,1\right\}$ and $P^{\prime \prime }\to W_{+}$ are the initial marking functions, where:*

   *$M_{0}\left(p_{j}^{\prime \prime }\right)=z_{j}K\left(p_{j}^{\prime \prime }\right)$,*

   *$z_{j}\in ℵ\cup \left\{0\right\}$,*

   *$z_{j}\leq K\left(p_{j}^{\prime \prime }\right)$,*

   *$1\leq j\leq a^{\prime \prime }$,*

   *$W_{+}$ is a set of non-negative rational numbers;*

*e is an event that synchronizes the work of all transitions*.

**Figure 1:**
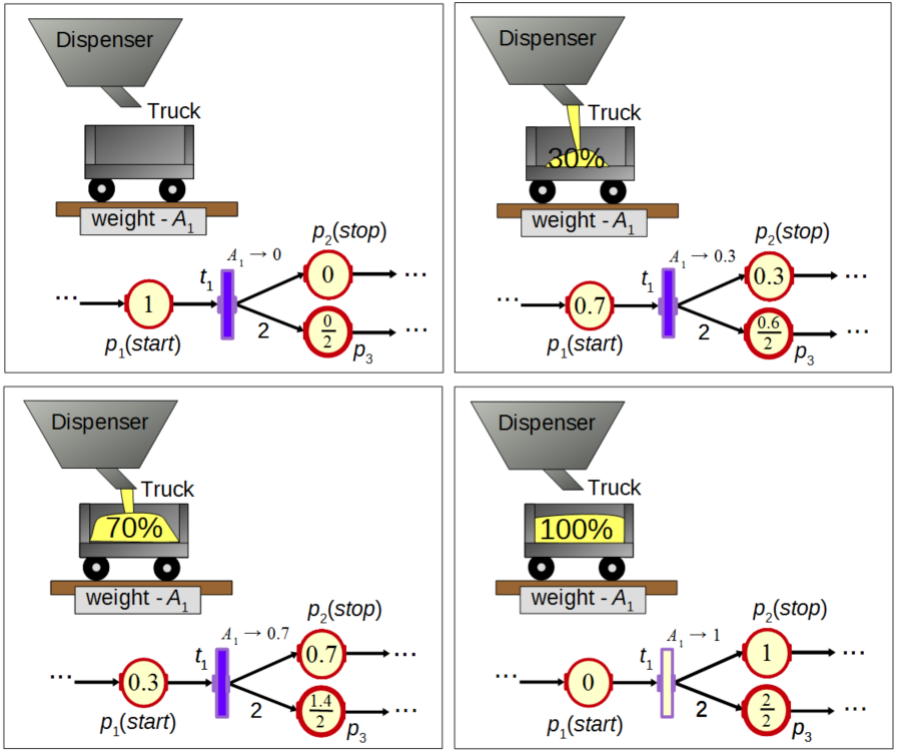
An algorithm in the form of a FIPN graph to control the dispensing process.

FIPN can be represented as a bipartite graph. An exemplary net and a system controlled by it (in different states) are presented in Figure 1. There exist two types of places in the net: $p^{\prime }$-type—$p_{1}$ and $p_{2}$ (for process modeling) and $p^{\prime \prime }$-type—$p_{3}$ (for resource modeling). They are shown as circles. For both types, the marking is a real number in the range [0, 1] located inside the circle. However, the marking of $p^{\prime \prime }$-type places is presented as a fraction and can hold more than one token. The denominator holds the capacity of a place, while the numerator holds the number of tokens.

Moreover, statements can be assigned to $p^{\prime }$-type places to set the value of process variables. In Figure 1 actions start and stop are assigned to places $p_{1}$ and $p_{2}$ for the control of a dispensing process. Transitions are represented by rectangles and can be related to binary or analogue signals. Transition $t_{1}$ is synchronized by an analogue signal $A_{1}$ representing the weight of the material transferred to the truck (Figure 1). In FIPN, analogue signals are normalized into the range [0, 1]. As can be seen in the figure, the transfer of a token across the fired transition is a process with a duration longer than one clock cycle which synchronizes the net operation. This duration depends on the increment of the sensor value. Such operation of transitions allows for better visualization as well as for more precise control in comparison to discrete PNs. Additionally, some logic conditions (an example is shown in Figure 3) can be assigned to transitions.

The basic conditions for transition activation and deactivation are described in Definition 2.

**Definition** **2.** *The transition t∈t with marking M:P→[0,1] is enabled when the degree to fulfill the condition Θ(t)=θ, assigned to the transition, is greater than zero and the following conditions are satisfied [59]:*

(1) $$\forall p\in {}^{•}t,M\left(p\right)\geq W\left(p,t\right)/K\left(p\right)$$

*and*

(2) $$\forall p\in t^{•},M\left(p\right)\leq 1-W\left(t,p\right)/K\left(p\right),$$

*until:*

(3) $$\exists p^{\prime }\in {}^{•}t,M\left(p^{\prime }\right)=0$$

*or*

(4) $$\exists p^{\prime }\in t^{•},M\left(p^{\prime }\right)=1,$$

*where:*

   *${}^{•}t=\left\{p\in P|\left(p,t\right)\in R\right\}$ is the set of input places of t*,

   *$t^{•}=\left\{p\in P|\left(t,p\right)\in R\right\}$ is the set of output places of t*.

The transfer of tokens from input to output places across the transition can begin when conditions (1) and (2) of Definition 2 are satisfied, while it ends when condition (3) or (4) is fulfilled.

The change of the marking for places connected to the enabled transition depends on the increment of the degree to which the condition corresponding to the transition is satisfied. The method for calculating the new marking is described by Definition 3. The transition remains active until the tokens are transferred from the input places to the output places.

**Definition** **3.** *Let M be the marking for which the transition t∈T is enabled, the degree Θ(t)=θ (where θ∈[0,1]), to which the condition corresponding to the enabled transition is satisfied, will be changed by Δθ≥0 and there will be an event e synchronizing the work of all transitions. The new marking of the net $M^{\prime }$ can be computed by the following rule [59]:*

(5) $$M^{\prime }\left(p\right)=\left\{\begin{array}{c} M(p)-\Delta \theta \cdot \frac{W(p,t)}{K(p)}\;\mathrm{for}\;p\in {}^{•}t\backslash t^{•},\\ M(p)+\Delta \theta \;\cdot \frac{W(t,p)}{K(p)}\;\mathrm{for}\;p\in \;t^{•}\backslash {}^{•}t,\\ M(p)-\Delta \theta \;\cdot \frac{W(p,t)-W(t,p)}{K(p)}\;\mathrm{for}\;p\in \;t^{•}\cap {}^{•}t,\\ M\left(p\right)\;\mathrm{for}\;p\notin \;t^{•}\cup {}^{•}t. \end{array}\right.$$

*The increment Δθ<0 does not introduce any changes in the net marking.*

**Figure 2:**
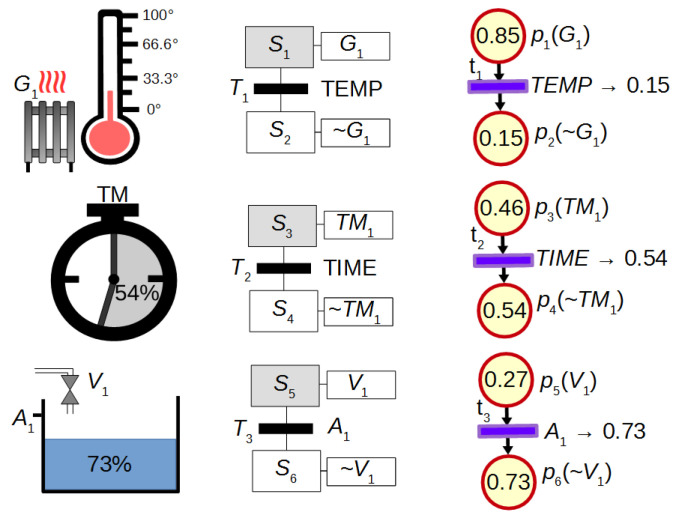
The advantage of FIPN visualization in comparison to discrete PNs.

It has been mentioned that FIPN gives better visualization effect in comparison to discrete PNs. This advantage can be seen in Figure 2 which presents the discrete PN graph and the FIPN graph (in different states) used for the control of heating, measuring time and filling the tank. Discrete PN displays only the actions that are currently performed, while FIPN additionally presents the progress of these actions.

**Figure 3:**
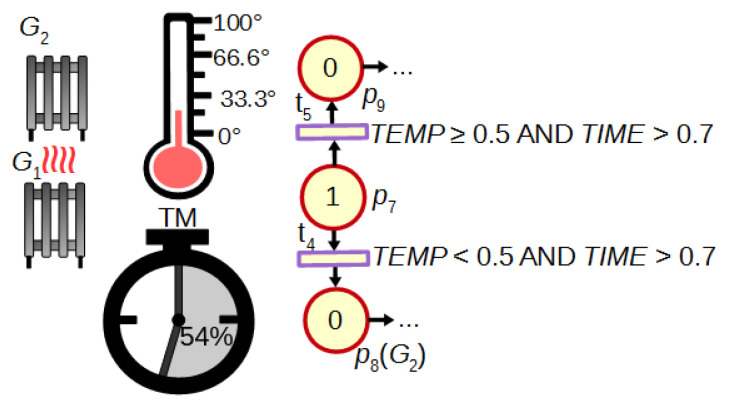
A dynamic control of heating.

Another advantage of FIPN in comparison to discrete PNs is the possibility of the dynamic control in response to changing values of analogue signals or fuzzy marking of places. An example of such control is shown in Figure 3. If the temperature is too low after 70% of the measured time (logic condition assigned to transition $t_{4}$), the additional heater $G_{2}$ is turned on (place $p_{8}$). If the temperature increases with enough speed ($t_{5}$), no action is needed ($p_{9}$). Such dynamic control is also presented in Section 4 (macroplace $mp_{4}$).

## 3. New HFIPN Concept and Its Definitions

In this section, a new concept and definitions of HFIPN are presented. The proposed net enables the creation of subnets with multiple input, output, and input-output places as well as the use of macroplace instances. Moreover, based on a HFIPN graph, a hierarchy graph is created. All of these functionalities allow for good adaptation of HFIPN to practical needs. The way of summing subnets and the conversion of HFIPN to its flat version are also described. Finally, a new algebraic representation and a brief introduction of HFIPN-SML features are given.

### 3.1. The Concept of HFIPN

**Figure 4:**
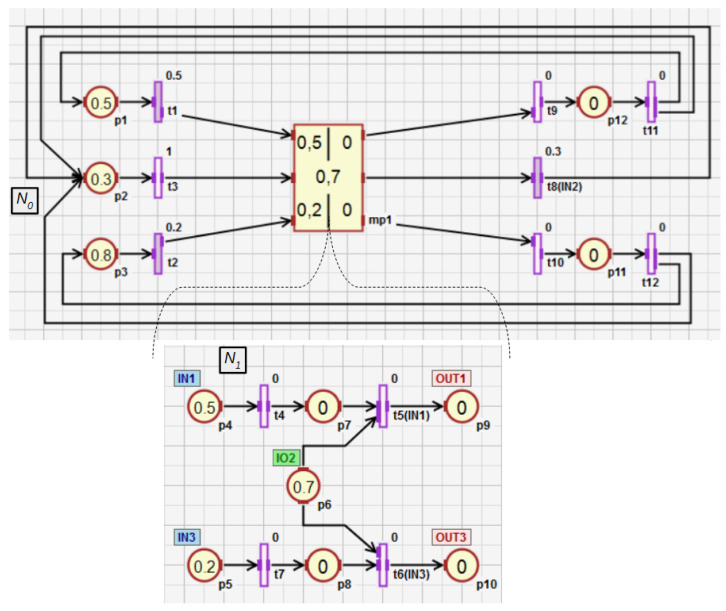
HFIPN graph with the macroplace having two input places, two output places and one input-output place.

In this subsection, the conception of HFIPN is presented. Figure 4 shows an example of the hierarchical net consisting of two subnets $N_{0}$ and $N_{1}$. The subnet $N_{0}$ contains the macroplace $MP_{1}$ which includes the subnet $N_{1}$. The macroplace $Mp_{1}$ is an internal macroplace of the subnet $N_{0}$ and is overriding for the subnet $N_{1}$. In the hierarchical net, a macroplace can have several input, output, and input-output places that are visible in its overriding and internal subnets. Input places can only have input arcs in the overriding subnet of a macroplace, while output places can only have output arcs. Input-output places can have both types of arcs in the overriding subnet. The macroplace $Mp_{1}$ (Figure 4) contains two input places ($p_{4}$ and $p_{5}$), two output places ($p_{9}$ and $p_{10}$), and one input-output place ($p_{6}$). The place $p_{6}$ has an input and output arc in the subnet $N_{0}$ and two output arcs in the subnet $N_{1}$. In the subnet $N_{1}$, the place $p_{6}$ can also have an input arc.

HFIPN enables the application of macroplace instances, which facilitates the use of modularization in a net. This functionality is analogous to that used in CPN [31]. It allows creating several macroplaces (subnets) that have the same structure: they have the same number of places with the same capacity, the same number of transitions and arcs of the same weights connecting the corresponding nodes. In the proposed concept, macroplaces that are instances of the same type do not share nodes and arcs. Therefore, the places, transitions, and arcs of one macroplace are always separate from the elements of other macroplaces, and in the algebraic representation, they are represented by different numbers in all vectors and an incidence matrix. However, the concept of macroplace instances allows for simultaneous modification of macroplaces of the same type.

**Figure 5:**
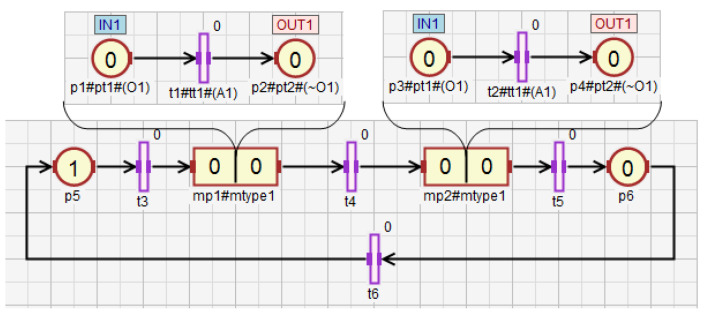
The net with two macroplaces of the same type and shared input and output variables.

**Figure 6:**
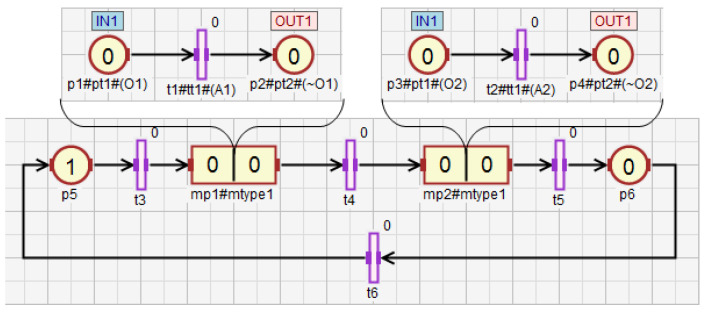
The net with two macroplaces of the same type, in which input and output variables are not shared.

**Figure 7:**
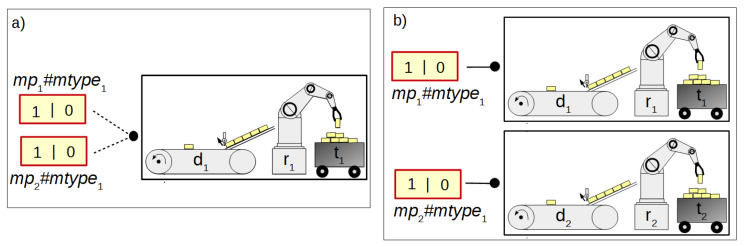
The application of two concepts of macroplace instances: (**a**) shared variables, (**b**) unshared variables.

The concept of macroplace instances entails the introduction of place, transition and arc instances, but only for those elements that are inside macroplaces. Places that are instances of the same type may be associated with the same statements, while transitions may be associated with the same conditions. Therefore, instances of places and transitions belonging to macroplace instances can be created in two ways in HFIPN. In the first approach, the statements assigned to the places and conditions associated with the transitions are shared between nodes of the same type, while in the second approach they are separate. In Figure 5 the first type of place and transition instances is presented, in which statements and conditions are shared. There are two macroplaces $Mp_{1}$ and $Mp_{2}$ in the net, that are of the same type $mtype_{1}$. The places $p_{1}$ and $p_{2}$ from the subnet $N_{1}$ are functionally the same places as $p_{3}$ and $p_{4}$ from the subnet $N_{2}$. They set the variable $O_{1}$. The same applies to the transitions $t_{1}$ and $t_{2}$, whose degrees of the conditions fulfillment are synchronized with the variable $A_{1}$. In Figure 6, the second type of macroplace instances with separate statements and conditions is presented. The macroplaces $Mp_{1}$ and $Mp_{2}$ do not share the output variables assigned to places. The places $p_{1}$ and $p_{2}$ set the variable $O_{1}$, and the places $p_{3}$ and $p_{4}$ set $O_{2}$. The variables synchronizing the degrees to which the conditions corresponding to the transitions are satisfied for transitions $t_{1}$ (variable $A_{1}$) and $t_{2}$ ($A_{2}$) are also not shared between macroplaces. The concept of macroplace instances with shared variables can be used when a certain part of a system is controlled by several modules in the same way—Figure 7a. The second approach with unshared variables is used when similar parts of a system are controlled in the same way by different macroplaces—Figure 7b.

### 3.2. Formal Description of HFIPN

Three definitions specify the structure and operation of HFIPN. The first of them, the main definition of a whole net, is as follows.

**Definition** **4.** *The hierarchical FIPN is the system:*

*HFIPN =(N,MP,IO,INS), where:*

*$N=\{N_{0},N_{1},...,N_{d}\}$ is a nonempty finite set of subnets described in Definitions 5 and 6;*

*$MP=\{mp_{1},mp_{2},...,mp_{d}\}$ is an finite set of macroplaces, such that $\forall mp_{i}\in MP$:*

  *$mp_{i}=\left\{N_{i},P_{mp(i)}\right\}$, where:*

  *$N_{i}\in N\left(1\leq i\leq d\right)$ is a subnet, for which the macroplace $mp_{i}$ is overriding (the subnet*

  *$N_{i}$ is internal for the macroplace $mp_{i}$);*

  *$P_{mp(i)}=P_{mp(i)}^{in}\cup P_{mp(i)}^{out}\cup P_{mp(i)}^{io}$ is a nonempty finite set of input,*

    *output and input-output places of a macroplace;*

*IO:P→{in,out,iNout,NormaL} is a function determining the place type;*

*INS is the function that returns the instance type for any node or arc:*

  $INS=\left\{\begin{array}{c} MP\to MTYPE,\\ P\to PTYPE,\\ T\to TTYPE,\\ R\to RTYPE, \end{array}\right.$

  *where:*

  *$MTYPE=\{mtype_{1},mtype_{2},...\}$ is a set of instance types of macroplaces,*

  *$PTYPE=\{ptype_{1},ptype_{2},...\}$ is a set of instance types of places,*

  *$TTYPE=\{ttype_{1},ttype_{2},...\}$ is a set of instance types of transitions,*

  *$RTYPE=\{rtype_{1},rtype_{2},...\}$ is a set of instance types of arcs*.

By Definition 4, each subnet except $N_{0}$ has exactly one overriding macroplace. Moreover, HFIPN is divided into d+1 subnets. If d=0, the net is flat, and if d>0, the net is hierarchical. The subnet $N_{0}$ is the main or top level subnet. The remaining subnets are called lower level subnets or macronets. Definition 4 also shows that the elements of a macroplace are sets of input, output, and input-output places. To distinguish the types of places in the net, the global function IO was added to the main definition, which allows specifying the type of each place in the net, whether it is an input, output, input and output of a macroplace or a normal place. Each macroplace in HFIPN is an instance of a given type. The remaining elements (places, transitions, and arcs) are instances if they occur inside macroplaces (e.g., the place $p_{1}$ in Figure 5 is an instance of type $pt_{1}$). However, the elements are not instances if they occur directly in the main subnet $N_{0}$ (e.g., the place $p_{5}$ in Figure 5).

Definition 5 describes the structure of each subnet in HFIPN.

**Definition** **5.** *Each subnet $N_{i},0\leq i\leq d$ is defined as follows:*

*$N_{i}\;=\;\left(MP_{i},P_{i},T_{i},\Omega_{i},\Psi_{i},R_{i},\Delta_{i},K_{i},W_{i},\Gamma_{i},\Theta_{i},M_{0(i)},e\right)$, where:*

*$MP_{i}\subseteq MP$ is a finite set of internal macroplaces of the subnet $N_{i}$ ($N_{i}$ is an overriding for these*

     *macroplaces);*

*$P_{i}\subseteq P$ and $P_{i}=P_{i}^{\prime }\cup P_{i}^{\prime \prime }\cup P_{i}^{in}\cup P_{i}^{out}\cup P_{i}^{io}$ is a nonempty finite set of places (in the subnet*

     *$N_{i}$), where:*

     *$P_{i}^{\prime }\subseteq P^{\prime }$ is a set of places of the type $p^{\prime }$,*

     *$P_{i}^{\prime \prime }\subseteq P^{\prime \prime }$ is a set of places of the type $p^{\prime \prime }$,*

     *$P_{mp(i)}\subseteq P_{i}^{\prime }\cup P_{i}^{\prime \prime }$ for i≠0,*

     *$P_{i}^{in}=P_{i}^{{}^{\prime }in}\cup P_{i}^{{}^{\prime \prime }in}$ is a set of input places (of the type $p^{\prime }$ or $p^{\prime \prime }$) of internal macroplaces*

          *from the set $MP_{i}$,*

     *$P_{i}^{out}=P_{i}^{{}^{\prime }out}\cup P_{i}^{{}^{\prime \prime }out}$ is a set of output places (of the type $p^{\prime }$ or $p^{\prime \prime }$) of internal macroplaces*

          *from the set $MP_{i}$,*

     *$P_{i}^{io}=P_{i}^{{}^{\prime }io}\cup P_{i}^{{}^{\prime \prime }io}$ is a set of inputoutput places (of the type $p^{\prime }$ or $p^{\prime \prime }$) of internal*

          *macroplaces from the set $MP_{i}$,*

     *$P_{i}^{\prime }$, $P_{i}^{\prime \prime }$, $P_{i}^{{}^{\prime }in}$, $P_{i}^{{}^{\prime \prime }in}$, $P_{i}^{{}^{\prime }out}$, $P_{i}^{{}^{\prime \prime }out}$, $P_{i}^{{}^{\prime }io}$, $P_{i}^{{}^{\prime \prime }io}$ any two of them have no common elements;*

*$T_{i}\subseteq T$ is a nonempty finite set of transitions;*

*$\Omega_{i}\subseteq \Omega$ is a nonempty finite set of statements;*

*$\Psi_{i}\subseteq \Psi$ is a nonempty finite set of conditions;*

*$R_{i}\subseteq R$ and $R_{i}\subseteq \left(\left(P_{i}^{\prime }\cup P_{i}^{\prime \prime }\cup P_{i}^{out}\cup P_{i}^{io}\right)\times T_{i}\right)\cup (T_{i}$$\times (P_{i}^{\prime }\cup P_{i}^{\prime \prime }\cup$$P_{i}^{in}\cup P_{i}^{io}))$ is a subnet*

     *incidence relation;*

*$\Delta_{i}:P_{i}\to \Omega_{i}$ is the function assigning a statement to each place;*

*$K_{i}:\left(P_{i}^{\prime }\cup P_{i}^{{}^{\prime }in}\cup P_{i}^{{}^{\prime }out}\cup P_{i}^{{}^{\prime }io}\right)\to \left\{1\right\}$ and $(P_{i}^{\prime \prime }\cup P_{i}^{{}^{\prime \prime }in}\cup$$P_{i}^{{}^{\prime \prime }out}\cup P_{i}^{{}^{\prime \prime }io})\to$ℵ\{1} are the*

     *functions assigning a capacity to each place;*

*$\Gamma_{i}:T_{i}\to \Psi_{i}$ is the function assigning a condition to each transition;*

*$\Theta_{i}:T_{i}\to \left[0,1\right]$ is the function defining the degree to which the condition corresponding to the*

     *transitions is satisfied;*

*$W_{i}:R_{i}\to ℵ$ is the weight function, which for $\forall p\in P_{i}$ and $\forall t\in T_{i}$ meets two conditions:*

     *$W_{i}\left(p,t\right)\leq K_{i}\left(p\right)$,*

     *$W_{i}\left(t,p\right)\leq K_{i}\left(p\right)$;*

*$M_{0(i)}:\left(P_{i}^{\prime }\cup P_{i}^{{}^{\prime }in}\cup P_{i}^{{}^{\prime }out}\cup P_{i}^{{}^{\prime }io}\right)\to \left\{0,1\right\}$ and $(P_{i}^{\prime \prime }\cup$$P_{i}^{{}^{\prime \prime }in}\cup P_{i}^{{}^{\prime \prime }out}\;\cup P_{i}^{{}^{\prime \prime }io})\to W_{+}$ are the*

     *initial marking functions, where for each $p^{\prime \prime }\in \left(P_{i}^{\prime \prime }\cup P_{i}^{{}^{\prime \prime }in}\cup P_{i}^{{}^{\prime \prime }out}\cup P_{i}^{{}^{\prime \prime }io}\right)$ exists*

     *z∈ℵ∪{0}, such that:*

     *$z\leq K_{i}\left(p^{\prime \prime }\right)$,*

     *$M_{0(i)}\left(p^{\prime \prime }\right)=zK_{i}\left(p^{\prime \prime }\right)$;*

*e is the global event which synchronizes the work of all transitions.*

By Definition 5 each set of places $P_{i}$, the transitions $T_{i}$, the conditions $\Psi_{i}$, the statements $\Omega_{i}$ and the arcs $R_{i}$, which belongs to the subnet $N_{i}$, is a subset of the flat net set: *P*, *T*, Ψ, Ω and *R* respectively. From Definitions 4 and 5, it follows that the main subnet may have internal macroplaces, which are overriding macroplaces for some subnet, which, in turn, may contain further macroplaces, etc. The level of maximum depth in HFIPN is not limited in the definition. Subnets/macroplaces only in a special case may have common elements (places) with other subnets/macroplaces, which is described in Definition 6 specifying the relationship between sets of subnets in HFIPN.

**Definition** **6.** *For any two subnets $N_{i}$, $N_{j}\in N$ for 0≤i≤d, 0≤j≤d and i≠j the relationship between the subsets of places, transitions, statements, macroplaces, conditions assigned to transitions, arcs is defined as follows:*

(6) $$P_{i}\cap P_{j}=\left\{\begin{array}{c} P_{mp(i)}\;\mathrm{for}\;mp_{i}\in MP_{j}\;\mathrm{and}\;i\neq 0,\\ P_{mp(j)}\;\mathrm{for}\;mp_{j}\in MP_{i}\;\mathrm{and}\;j\neq 0,\\ ⌀\;\mathrm{in}\;\mathrm{other}\;\mathrm{cases},\; \end{array}\right.$$

(7) $$T_{i}\cap T_{j}=⌀,$$

(8) $$\Omega_{i}\cap \Omega_{j}=⌀,$$

(9) $$\Psi_{i}\cap \Psi_{j}=⌀,$$

(10) $$R_{i}\cap R_{j}=⌀,$$

(11) $$MP_{i}\cap MP_{j}=⌀,$$

*and for additionally fulfilled conditions d>1 and i,j≠0*:

(12) $$P_{mp(i)}\cap P_{mp(j)}=⌀.$$

Definition 6 shows that for any two subnets, for which the overriding macroplace of one of them is the internal macroplace of the other, the common part is the set of input, output and input-output places of this macroplace (Equation (6)). The example of such a set in Figure 4 is the set $P_{mp(1)}=\left\{p_{4},p_{5},p_{6},p_{9},p_{10}\right\}$. Places from this set belong to the subnets $N_{0}$ and $N_{1}$. However, the introduced restrictions do not allow the input and output place of the first macroplace/subnet to be the input and output place of the second macroplace/subnet (Equation (12)). This prevents the possibility of the creation of an infinite number of macroplaces for a finite number of places.

### 3.3. The Hierarchy in HFIPN

This subsection presents a hierarchy graph, allowing to define nesting relations between macroplaces (subnets) in HFIPN and facilitates navigation to various areas of the net. The terms defining the degree of the nesting relation: ancestor, direct ancestor, descendant, and direct descendant are also introduced. The special function ∗() is used to formulate the concept of ancestor and descendant, and in the next subsection to define an algebraic representation.

First, the function ∗() is introduced. For each subnet $N_{i}\in N$, $N_{i}=(MP_{i},P_{i},T_{i},\Omega_{i},$$\Psi_{i},$$R_{i},$$\Delta_{i},$$K_{i},$$W_{i},$$\Gamma_{i}$, $\Theta_{i},M_{0(i)}$, e) the function $*(N_{i})$, abbreviated $*N_{i}$, is defined as follows:

(13) $$*N_{i}=\left\{\begin{array}{c} N_{i}\;\mathrm{for}\;MP_{i}=⌀,\\ N_{i}\cup \sum_{\forall mp_{j}\in MP_{i}}*N_{j}\;\mathrm{otherwise}. \end{array}\right.$$

Definition 7 specifies what is the sum of two subnets.

**Definition** **7.** *For any two subnets $N_{i},N_{j}\in N$ for 0≤i≤d, 0≤j≤d and i≠j their sum $N_{k}$ is calculated as follows:*

*$N_{k}=N_{i}\cup N_{j}=(MP_{k},P_{k},T_{k},\Omega_{k},\Psi_{k},R_{k},\Delta_{k},$$K_{k},$$W_{k},$$\Gamma_{k},$$\Theta_{k},M_{0(k)},e),$ where:*

(14) $$MP_{k}=\left\{\begin{array}{c} \left(MP_{i}\cup MP_{j}\right)\backslash \left\{mp_{i}\right\}\;\mathrm{for}\;mp_{i}\in MP_{j}\;\mathrm{and}\;i\neq 0,\\ \left(MP_{i}\cup MP_{j}\right)\backslash \left\{mp_{j}\right\}\;\mathrm{for}\;mp_{j}\in MP_{i}\;\mathrm{and}\;j\neq 0,\\ MP_{i}\cup MP_{j}\;\mathrm{in}\;\mathrm{the}\;\mathrm{other}\;\mathrm{cases}; \end{array}\right.$$

(15) $$P_{k}=P_{k}^{\prime }\cup P_{k}^{\prime \prime }\cup P_{k}^{in}\cup P_{k}^{out}\cup P_{k}^{io},where:$$

(16) $$P_{k}^{\prime }=P_{i}^{\prime }\cup P_{j}^{\prime },$$

(17) $$P_{k}^{\prime \prime }=P_{i}^{\prime \prime }\cup P_{j}^{\prime \prime },$$

(18) $$P_{k}^{in}=\left\{\begin{array}{c} \left(P_{i}^{in}\cup P_{j}^{in}\right)\backslash P_{mp(i)}^{in}\mathrm{for}mp_{i}\in MP_{j}\mathrm{and}i\neq 0,\\ \left(P_{i}^{in}\cup P_{j}^{in}\right)\backslash P_{mp(j)}^{in}\mathrm{for}mp_{j}\in MP_{i}\mathrm{and}j\neq 0,\\ P_{i}^{in}\cup P_{j}^{in}\mathrm{in}\mathrm{the}\mathrm{other}\mathrm{cases}, \end{array}\right.$$

(19) $$P_{k}^{out}=\left\{\begin{array}{c} \left(P_{i}^{out}\cup P_{j}^{out}\right)\backslash P_{mp(i)}^{out}\;\mathrm{for}\;mp_{i}\in MP_{j}\;\mathrm{and}\;i\neq 0,\\ \left(P_{i}^{out}\cup P_{j}^{out}\right)\backslash P_{mp(j)}^{out}\;\mathrm{for}\;mp_{j}\in MP_{i}\;\mathrm{and}\;j\neq 0,\\ P_{i}^{out}\cup P_{j}^{out}\mathrm{in}\mathrm{the}\mathrm{other}\mathrm{cases}, \end{array}\right.$$

(20) $$P_{k}^{io}=\left\{\begin{array}{c} \left(P_{i}^{io}\cup P_{j}^{io}\right)\backslash P_{mp(i)}^{io}\;\mathrm{for}\;mp_{i}\in MP_{j}\;\mathrm{and}\;i\neq 0,\\ \left(P_{i}^{io}\cup P_{j}^{io}\right)\backslash P_{mp(j)}^{io}\;\mathrm{for}\;mp_{j}\in MP_{i}\;\mathrm{and}\;j\neq 0,\\ P_{i}^{io}\cup P_{j}^{io}\mathrm{in}\mathrm{the}\mathrm{other}\mathrm{cases}; \end{array}\right.$$

(21) $$T_{k}=T_{i}\cup T_{j};$$

(22) $$\Omega_{k}=\Omega_{i}\cup \Omega_{j};$$

(23) $$\Psi_{k}=\Psi_{i}\cup \Psi_{j};$$

(24) $$R_{k}=R_{i}\cup R_{j};$$

(25) $$\Delta_{k}:P_{k}\to \Omega_{k};$$

(26) $$K_{k}:\left(P_{k}^{\prime }\cup P_{k}^{{}^{\prime }in}\cup P_{k}^{{}^{\prime }out}\cup P_{k}^{{}^{\prime }io}\right)\to 1\;\mathrm{and}\;\left(P_{k}^{{}^{\prime \prime }}\cup P_{k}^{{}^{\prime \prime }in}\cup P_{k}^{{}^{\prime \prime }out}\cup P_{k}^{{}^{\prime \prime }io}\right)\to ℵ\backslash \left\{1\right\};$$

(27) $$\Gamma_{k}:T_{k}\to \Psi_{k};$$

(28) $$\Theta_{k}:T_{k}\to \left[0,1\right];$$

(29) $$W_{k}:R_{k}\to ℵ;$$

(30) $$M_{0(k)}:\left(P_{k}^{\prime }\cup P_{k}^{{}^{\prime }in}\cup P_{k}^{{}^{\prime }out}\cup P_{k}^{{}^{\prime }io}\right)\to \left\{0,1\right\}\;\mathrm{and}\;\left(P_{k}^{{}^{\prime \prime }}\cup P_{k}^{{}^{\prime \prime }in}\cup P_{k}^{{}^{\prime \prime }out}\;\cup P_{k}^{{}^{\prime \prime }io}\right)\to W_{+}.$$

By Definition 7, the sum of the subnets $N_{i}$ and $N_{j}$ is the subnet $N_{k}$, which consists of subsets (transitions, statements, conditions, and arcs), which are the sums of the corresponding subsets from the subnets $N_{i}$ and $N_{j}$. The subsets of macroplaces and places can be summed in a different way. If, for instance, the macroplace $mp_{i}$ is internal for the subnet $N_{j}$, then $mp_{i}$ is removed from the result subset of internal macroplaces of $N_{k}$ ($Mp_{k}$). Similar operations occur for the set of places $P_{k}$ (if $mp_{i}$ is internal for $N_{j}$). The input places $P_{mp(i)}^{in}$, the output places $P_{mp(i)}^{out}$ and the input-output places $P_{mp(i)}^{io}$ of $mp_{i}$ are removed from the sets $P_{k}^{in}$, $P_{k}^{out}$ and $P_{k}^{io}$, respectively. These places are still the elements of the normal place sets of the subnet $N_{k}$, i.e., $P_{k}^{\prime }\cup P_{k}^{\prime \prime }$.

In the HFIPN-SML simulator, there is no need to continuously add subnets to create the algebraic representation described in Section 3.4. For each subnet, the vectors and the incidence matrix are updated on-the-fly when places, transitions, and arcs are added to the subnet. The summation operation is used when a user wants to undo adding a new macroplace based on a selected fragment of the subnet.

Definition 7 and the function (13) allow for the transformation of a hierarchical net (HFIPN) into its flat equivalent (FIPN). This conversion involves the addition of all subnets of the hierarchical net. Some sets of the obtained net, i.e., the set of internal macroplaces and the sets of input, output, and input-output places of the internal macroplaces are empty:

(31) $$FIPN=*N_{0}$$

Based on Definition 7, the following functions for subsets of subnets can be created: $*(N_{i},P_{i})$, $*(N_{i},MP_{i})$, $*(N_{i},T_{i})$, $*(N_{i},\Omega_{i})$, $*(N_{i},\Psi_{i})$ and $*(N_{i},R_{i})$ (abbreviated $*P_{i}$, $*MP_{i}$, $*T_{i}$, $*\Omega_{i}$, $*\Psi_{i}$ and $*R_{i}$). They are defined as follows:

(32) $$*P_{i}=\left\{\begin{array}{c} p_{i}\;\mathrm{for}\;MP_{i}=⌀,\\ P_{i}\cup \sum_{\forall mp_{j}\in MP_{i}}*P_{j}\;\mathrm{otherwise}, \end{array}\right.$$

(33) $$*MP_{i}=\left\{\begin{array}{c} MP_{i}\;\mathrm{for}\;MP_{i}=⌀,\\ MP_{i}\cup \sum_{\forall mp_{j}\in MP_{i}}*MP_{j}\;\mathrm{otherwise}, \end{array}\right.$$

(34) $$*T_{i}=\left\{\begin{array}{c} T_{i}\;\mathrm{for}\;MP_{i}=⌀,\\ T_{i}\cup \sum_{\forall mp_{j}\in MP_{i}}*T_{j}\;\mathrm{otherwise}, \end{array}\right.$$

(35) $$*\Omega_{i}=\left\{\begin{array}{c} \Omega_{i}\;\mathrm{for}\;MP_{i}=⌀,\\ \Omega_{i}\cup \sum_{\forall mp_{j}\in MP_{i}}*\Omega_{j}\;\mathrm{otherwise}, \end{array}\right.$$

(36) $$*\Psi_{i}=\left\{\begin{array}{c} \Psi_{i}\;\mathrm{for}\;MP_{i}=⌀,\\ \Psi_{i}\cup \sum_{\forall mp_{j}\in MP_{i}}*\Psi_{j}\;\mathrm{otherwise}, \end{array}\right.$$

(37) $$*R_{i}=\left\{\begin{array}{c} R_{i}\;\mathrm{for}\;MP_{i}=⌀,\\ R_{i}\cup \sum_{\forall mp_{j}\in MP_{i}}*R_{j}\;\mathrm{otherwise}. \end{array}\right.$$

Functions ∗() (Equations (32)–(37)) were defined for subsets of places, macroplaces, transitions, statements, conditions, and arcs. Their notation used without parentheses, e.g., $*P_{i}$ for $*(N_{i},P_{i})$ can be treated as the set returned by them, which simplifies their definition. After defining the functions ∗(), the formal definitions of nesting relations between subnets can be formulated.

**Definition** **8.** *The subnet $N_{i}$ ($N_{i}\in N$) is a direct ancestor for the subnet $N_{j}$ ($N_{j}\in N$) if the overriding macroplace $mp_{j}$ ($mp_{j}=\left\{N_{j},P_{mp(j)}\right\}$) of the subnet $N_{j}$ belongs to internal macroplaces of the subnet $N_{i}$ ($mp_{j}\in MP_{i}$).*

**Definition** **9.** *The subnet $N_{i}$ ($N_{i}\in N$) is a direct descendant for the subnet $N_{j}$ ($N_{j}\in N$) if the overriding macroplace $mp_{i}$ ($mp_{i}=\left\{N_{i},P_{mp(i)}\right\}$) of the subnet $N_{i}$ belongs to internal macroplaces of the subnet $N_{j}$ ($mp_{i}\in MP_{j}$).*

**Definition** **10.** *The subnet $N_{i}$ ($N_{i}\in N$) is a ancestor for the subnet $N_{j}$ ($N_{j}\in N$) if the overriding macroplace $mp_{j}$ ($mp_{j}=\left\{N_{j},P_{mp(j)}\right\}$) of the subnet $N_{j}$ belongs to the set $*MP_{i}$ created according to Equation (33).*

**Definition** **11.** *The subnet $N_{i}$ ($N_{i}\in N$) is a descendant for the subnet $N_{j}$ ($N_{j}\in N$) if the overriding macroplace $mp_{i}$ ($mp_{i}=\left\{N_{i},P_{mp(i)}\right\}$ of the subnet $N_{i}$ belongs to the set $*MP_{j}$ created according to Equation (33).*

**Figure 8:**
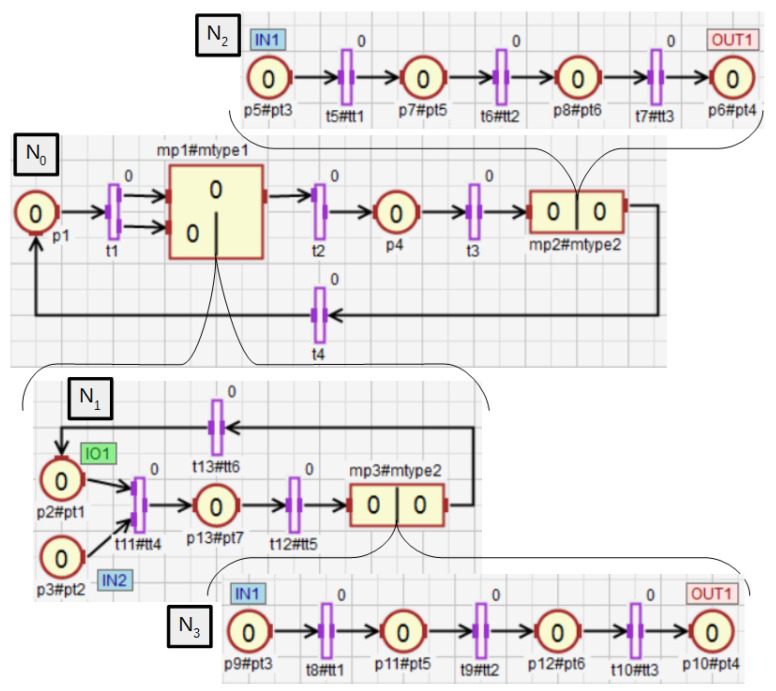
The net with three macroplaces, two of which are of the same type.

In Definitions 8–11, the nesting relations between subnets were defined. Nesting relations for macroplaces can be defined analogously. Therefore, the concepts of descendant, ancestor, direct descendant, and direct ancestor can also be used for macroplaces. The nesting relations and the function ∗() will be additionally shown on the example of a hierarchical net, which is presented in Figure 8. It contains three macroplaces. The macroplaces $mp_{1}$ and $mp_{2}$ belong to the subnet $N_{0}$, and the macroplace $mp_{3}$ belongs to the internal subnet of the macroplace $mp_{1}$. The macroplaces $mp_{2}$ and $mp_{3}$ are instances of the same type. For the subnet $N_{0}$ the set $*N_{0}$ has the form $N_{0}\cup N_{1}\cup N_{2}\cup N_{3}$, i.e., it contains this subnet and all of its descendant subnets. In turn, the direct descendants of the subnet $N_{0}$ are the subnets $N_{1}$ and $N_{2}$. A direct ancestor of the subnet $N_{3}$ is the subnet $N_{1}$, and all the ancestors of $N_{3}$ are subnets $N_{0}$ and $N_{1}$. The subnet $N_{3}$ has no descendants. For the main subnet $N_{0}$, the set $*P_{0}$ consists of all places in the net $\left\{p_{1},p_{2},\ldots ,p_{13}\right\}$, i.e., in the subnet $N_{0}$ and in its descendant subnets $N_{1}$, $N_{2}$ and $N_{3}$, while the set $*P_{3}$ has the form $\left\{p_{9},p_{10},p_{11},p_{12}\right\}$. The set $*t_{0}$ consists of all the transitions $\left\{t_{1},t_{2},\ldots ,t_{13}\right\}$ in the net, while $*t_{1}$ consists of the transitions $\left\{t_{8},t_{9},\ldots ,t_{13}\right\}$ from the subnet $N_{1}$ and all of its descendant subnets ($N_{3}$ in this case).

After defining the concepts related to the degree of nesting and function ∗(), the HFIPN hierarchy graph is formalized.

**Definition** **12.** *The hierarchy graph of HFIPN (HG_HFIPN) is a directed tree, such that:*

*$\mathrm{HG}\_\mathrm{HFIPN}=(mp_{0}^{hg},MP^{hg},R^{hg})$, where:*

*$mp_{0}^{hg}$ is a root of the tree representing the main subnet $N_{0}$ from Definition 4 marked as main;*

*$MP^{hg}=\left\{mp_{1}^{hg},mp_{2}^{hg},...,mp_{d}^{hg}\right\}$ is a set of remaining nodes of the tree representing macroplaces*

   *from the set MP from Definition 4;*

*$R^{hg}$ is a set of arcs connecting nodes, consisting of the elements such that*

   *$\forall mp_{i}^{hg},mp_{j}^{hg}\in MP^{hg}\cup \left\{mp_{0}^{hg}\right\}$, 0≤i,j≤d, i≠j exists the ordered pair of nodes*

   *$\left(mp_{i}^{hg},mp_{j}^{hg}\right)\in R^{hg}$ if and only if the macroplace $mp_{j}$ is an internal macroplace of the*

   *subnet $N_{i}$ ($mp_{j}\in MP_{i}$).*

**Figure 9:**
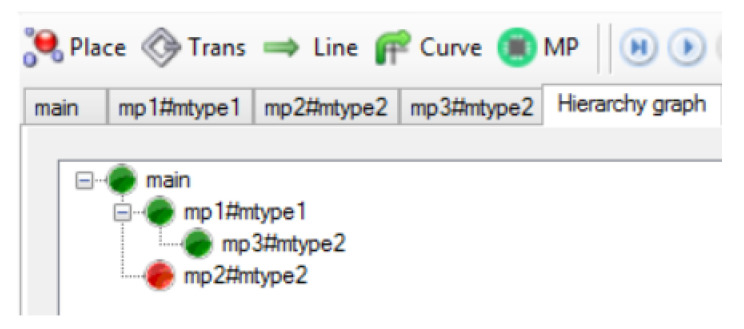
The hierarchy graph for the subnet from Figure 8.

A hierarchy graph is a directed graph in which nodes represent subnets (macroplaces) described in Definition 4. A hierarchy graph for the net from Figure 8 is shown in Figure 9. The graph allows for quick reading and understanding of subnets/macroplaces nesting, the number of instances and their nesting in HFIPN. A special node of the graph is the root representing the main subnet $N_{0}$, whose name is main since $N_{0}$ does not have an overriding macroplace. If there is an internal macroplace in a given subnet, the node representing it in the hierarchy graph is connected to the node representing the overriding macroplace for this subnet. The direction of the arcs is visible in the graphical representation by arranging the nodes. The node, to which the arc leads (e.g., $mp_{3}$ in Figure 9), is located below and to the right of the node ($mp_{1}$) which is the starting point of the arc. Arcs in the hierarchy graph are unlabeled. The advantage of the graph is the presentation of the hierarchical structure of the net: the level of depth and the indication of the way of macroplace nestings in the net. It also allows determining the number of instances of a given macroplace type, because the labels of nodes (except main) are represented by a pair consisting of the macroplace name and the instance type of this macro. Moreover, the software version of the hierarchy graph in the HFIPN-SML simulator allows a user to click on any node to move inside the macroplace (subnet) represented by that node.

### 3.4. The Algebraic Representation

In this subsection, the algebraic representation of HFIPN is presented. First, the auxiliary functions are defined and discussed:

(38) $$*\Delta_{i}:*P_{i}\to *\Omega_{i},$$

(39) $$\begin{array}{cc} \; & *K_{i}:\left(*P_{i}^{\prime }\cup *P_{i}^{{}^{\prime }in}\cup *P_{i}^{{}^{\prime }out}\cup *P_{i}^{{}^{\prime }io}\right)\to \left\{1\right\}\mathrm{and}\\ \; & \left(*P_{i}^{{}^{\prime \prime }}\cup *P_{i}^{{}^{\prime \prime }in}\cup *P_{i}^{{}^{\prime \prime }out}\cup *P_{i}^{{}^{\prime }io}\right)\to ℵ\backslash \left\{1\right\}. \end{array}$$

(40) $$*\Gamma_{i}:*T_{i}\to *\Psi_{i},$$

(41) $$*\Theta_{i}:*T_{i}\to \left[0,1\right],$$

(42) $$*W_{i}:*R_{i}\to ℵ,$$

(43) $$\begin{array}{cc} \; & *M_{0(i)}:\left(*P_{i}^{\prime }\cup *P_{i}^{{}^{\prime }in}\cup *P_{i}^{{}^{\prime }out}\cup *P_{i}^{{}^{\prime }io}\right)\to \left\{0,1\right\}\mathrm{and}\\ \; & \left(*P_{i}^{\prime \prime }\cup *P_{i}^{{}^{\prime \prime }in}\cup *P_{i}^{{}^{\prime \prime }out}\;\cup *P_{i}^{{}^{\prime \prime }io}\right)\to W_{+}. \end{array}$$

**Figure 10:**
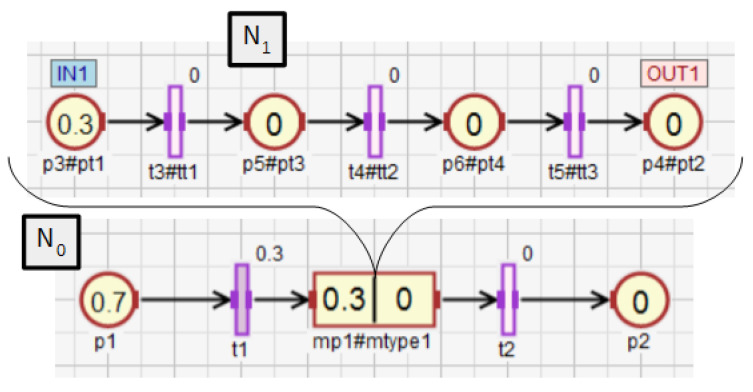
HFIPN with the single macroplace $mp_{1}$.

Functions (38)–(43) extend the following functions: assigning statements to places $\Delta_{i}\left(\right)$, returning the places capacity $K_{i}\left(\right)$, assigning conditions to transitions $\Gamma_{i}\left(\right)$, determining the degrees to which the conditions corresponding to the transitions $\Theta_{i}\left(\right)$, as well as weight functions $W_{i}\left(\right)$ and initial marking functions $M_{0(i)}\left(\right)$, which are all defined within a subnet (Definition 5). The extended functions $*\Delta_{i}\left(\right)$, $*K_{i}\left(\right)$, $*\Gamma_{i}\left(\right)$, $*\Theta_{i}\left(\right)$, $*W_{i}\left(\right)$, $*M_{0(i)}\left(\right)$ defined for a subnet $N_{i}$ can take as arguments elements that belong to $N_{i}$ directly or to its descendant subnets. These functions are explained using the exemplary net shown in Figure 10 and the function returning the capacity of places. The other extended functions work in the same way. For the subnet $N_{0}$, the function $K_{0}\left(\right)$ arguments can only be places that are directly in the subnet, i.e., $p_{1}$–$p_{4}$. However, for the same subnet, the function $*K_{0}\left(\right)$ can additionally return capacity for the places $p_{5}$ and $p_{6}$, i.e., all places of descendant subnets for $N_{0}$ ($N_{1}$ in this case). If the subnet has no descendant subnets ($N_{1}$ in Figure 10), then the functions $K_{i}\left(\right)$ and $*K_{i}\left(\right)$ work in the same way. The functions $K_{1}\left(\right)$ and $*K_{1}\left(\right)$ can only return capacity of the places $p_{3}$–$p_{6}$.

Each subnet $N_{i}$ in HFIPN may be represented by its own algebraic representation $N_{i}=(*C_{i},*\Omega_{i},*\Psi_{i},*\Delta_{i},*K_{i},$$*\Gamma_{i},*\Theta_{i},*M_{0(i)})$. The algebraic representation of the subnet $N_{0}$ is used to simulate the work of the entire net. However, for the remaining subnets, it can be used to test the properties or to simulate the work of the subnet, separately. The most important element of the algebraic representation is the incidence matrix $*C_{i}$ defined analogously to classical Petri nets, but taking the hierarchy into account. It has the following form.

**Definition** **13.** *Let HFIPN=(N,Mp,IO,INS) be the hierarchical fuzzy interpreted Petri net, and the subnet $N_{i}$ = $\{MP_{i}$, $P_{i}$, $T_{i}$, $\Omega_{i}$, $\Psi_{i}$, $R_{i}$, $\Delta_{i}$, $K_{i}$, $W_{i}$, $\Gamma_{i}$, $\Theta_{i}$, $M_{0(i)}$, e} one of its subnets ($N_{i}\in N$), for which $card\left(*T_{i}\right)=*b_{i}$ and $card\left(*P_{i}\right)=*a_{i}$. The incidence matrix $*C_{i}=*C_{i}^{+}-*C_{i}^{-}$ is defined as follows by the matrices: $*C_{i}^{+}=\left\{*c_{jk}^{+}\right\}$ and $*C_{i}^{-}=\left\{*c_{jk}^{-}\right\}$, both with a dimension $*b_{i}\times *a_{i}$:*

(44) $$*c_{jk}^{+}=\left\{\begin{array}{c} {}*W_{i}\left(t_{j},p_{k}\right)\;\mathrm{for}\;\left(t_{j},p_{k}\right)\in *R_{i},\\ 0\;\mathrm{for}\;\left(t_{j},p_{k}\right)\notin *R_{i}, \end{array}\right.$$

(45) $$*c_{jk}^{-}=\left\{\begin{array}{c} {}*W_{i}\left(p_{k},t_{j}\right)\;\mathrm{for}\;\left(p_{k},t_{j}\right)\in *R_{i},\\ 0\;\mathrm{for}\;\left(p_{k},t_{j}\right)\notin *R_{i}, \end{array}\right.$$

*for $j=1,2,...,*b_{i}$ and $k=1,2,...,*a_{i}$.*

The HFIPN algebraic representation enables the computation of a new marking in the net in response to transition activation and token movement between places. It uses vector and matrix operations, such as addition (46), multiplication (47), division (48), and minimum of two numbers (49):

(46) $$D={\left[d_{ij}\right]}_{n\times m}=A+B,\;d_{ij}=a_{ij}+b_{ij},$$

(47) $$E={\left[e_{ij}\right]}_{n\times m}=A\cdot B,\;e_{ij}=a_{ij}\cdot b_{ij},$$

(48) $$F={\left[f_{ij}\right]}_{n\times m}=\frac{A}{B},\;f_{ij}=\frac{a_{ij}}{b_{ij}},$$

(49) $$G={\left[g_{ij}\right]}_{n\times m}=A\wedge B,\;g_{ij}=a_{ij}\wedge b_{ij},$$

where: *i* = 1, 2, ..., *n* and *j* = 1, 2, ..., *m*.

Therefore, for each subnet $N_{i}\in N$, the algebraic representation takes the following form:

(50) $$*M_{i}^{\prime }=*M_{i}+\frac{*U_{i}\wedge \Delta *\Theta_{i}\cdot \left(*C_{i}^{+}-*C_{i}^{-}\right)}{*K_{i}},$$

where:

$*M_{i}$ is the vector of the length $1\times *a_{i}$, holding the current marking of places, where $*a_{i}=card\left(*P_{i}\right)$;
$*M_{i}^{\prime }$ is the vector of the length $1\times *a_{i}$, holding the new marking of places;
$*U_{i}$ is the vector of the length $1\times *b_{i}$, in which the given coefficient is equal to one if it corresponds to the enabled transition by the marking $*M_{i}$ in the subnet, where $*b_{i}=card(*T_{i}$);
$\Delta *\Theta_{i}$ is the vector of the length $1\times *b_{i}$, in which the coefficient $\Delta \theta_{j}$ ($1\leq j\leq *b_{i}$) describes the increment in the degree to which the condition corresponding to the transition $t_{j}$ ($t_{j}\in *T_{i}$) is satisfied;
$*K_{i}$ is the vector of the length $1\times *a_{i}$, holding the places capacity.

For the subnet $N_{0}$ from Figure 10, the incidence matrix $*C_{0}$ and the vectors $*M_{0}$, $*K_{0}$, $*U_{0}$, $*\Theta_{0}$ take the following form:

(51) $$*C_{0}=\begin{array}{c} \begin{array}{cccccc} \;p_{1} & p_{2} & p_{3} & \;p_{4} & \;p_{5} & \;p_{6} \end{array} \end{array},$$

(52) $$*M_{0}=\begin{array}{ccccccccc} \; & & p_{1} & p_{2} & p_{3} & p_{4} & p_{5} & p_{6}\\ \; & ( & 0.7 & 0 & 0.3 & 0 & 0 & 0 & ) \end{array},$$

(53) $$*K_{0}=\begin{array}{ccccccccc} & & p_{1} & p_{2} & p_{3} & p_{4} & p_{5} & p_{6}\\ \; & ( & 1 & 1 & 1 & 1 & 1 & 1 & ) \end{array},$$

(54) $$*U_{0}=\begin{array}{cccccccc} & & t_{1} & t_{2} & t_{3} & t_{4} & t_{5}\\ \; & ( & 1 & 0 & 0 & 0 & 0 & ) \end{array},$$

(55) $$*\Theta_{0}=\begin{array}{cccccccc} & & t_{1} & t_{2} & t_{3} & t_{4} & t_{5}\\ \; & ( & 0.3 & 0 & 0 & 0 & 0 & ) \end{array}.$$

If the increment of the degree to which the condition corresponding to the transition $t_{1}$ is satisfied changes by $\Delta \theta_{1}=0.1$, and the event *e*, synchronizing the work of all transition, occurs, then the vector $\Delta \Theta_{0}$ takes the following form:

(56) $$\Delta *\Theta_{0}=\begin{array}{cccccccc} & & t_{1} & t_{2} & t_{3} & t_{4} & t_{5}\\ \; & ( & 0.1 & 0 & 0 & 0 & 0 & ) \end{array}.$$

According to (50), the vector of the new marking $*M_{0}^{\prime }$ takes the following form:

(57) $$*M_{0}^{\prime }=\begin{array}{ccccccccc} & & p_{1} & p_{2} & p_{3} & p_{4} & p_{5} & p_{6}\\ \; & ( & 0.6 & 0 & 0.4 & 0 & 0 & 0 & ) \end{array}.$$

After that, the vector $*\Theta_{0}$ changes as follows:

(58) $$*\Theta_{0}=\begin{array}{cccccccc} & & t_{1} & t_{2} & t_{3} & t_{4} & t_{5}\\ \; & ( & 0.4 & 0 & 0 & 0 & 0 & ) \end{array},$$

and the vector $\Delta *\Theta_{0}$, which contains the increments of the degrees to which the conditions corresponding to the transitions are satisfied, is reset to zero at each calculation cycle. Therefore, the vector $*\Theta_{0}$ is used for storing the current values of these degrees and to calculate their increments at the beginning of each cycle.

### 3.5. HFIPN-SML Tool

In this subsection HFIPN-SML is briefly described. Most of the figures presented in this and the next section are created using screenshots from this application. Although the tool has not yet been published, it implements some functionalities that can be useful in practical modeling. In the current version, HFIPN-SML enables:

(a) the creation of a net graph,
(b) the use of a hierarchical structure including macroplace instances,
(c) the automatic code generation in Structured Text (ST) language for PLC controllers based on a non-hierarchical graph,
(d) the hierarchy graph presentation,
(e) displaying all vectors and matrices from algebraic representation,
(f) automatic and step simulations of a net operation,
(g) monitoring the operation of a program generated based on a non-hierarchical net.

All these features can make HFIPN-SML useful for practical modeling from the control system design to its practical implementation, and even then for monitoring purposes. However, some gaps need to be filled, i.e., automatic code generation module based on a hierarchical net and automatic analysis of properties integrated with HFIPN-SML.

## 4. Exemplary Application

**Figure 11:**
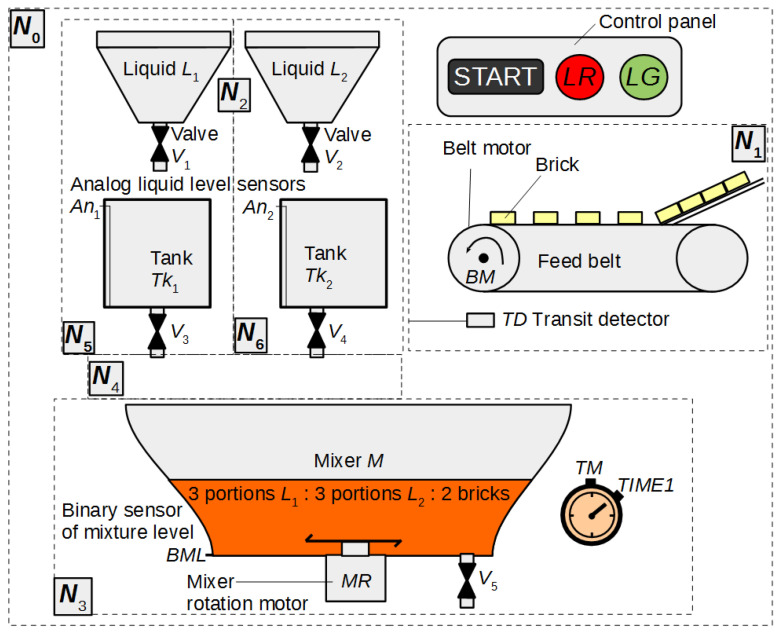
The scheme of the system to mix three components: two liquids and soluble bricks. Based on [64].

**Figure 12:**
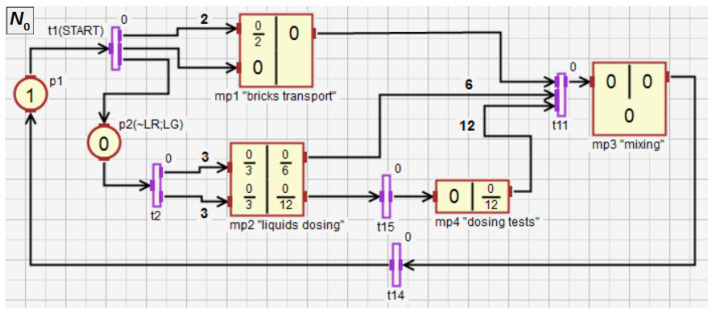
The main subnet of the control algorithm in the form of HFIPN graph.

**Figure 13:**
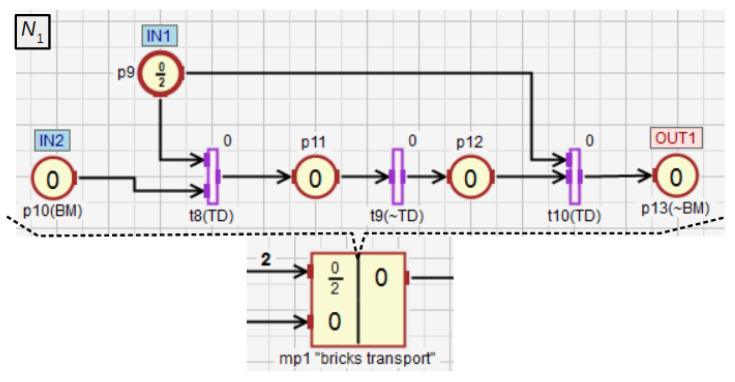
The brick transport module to the mixer.

**Figure 14:**
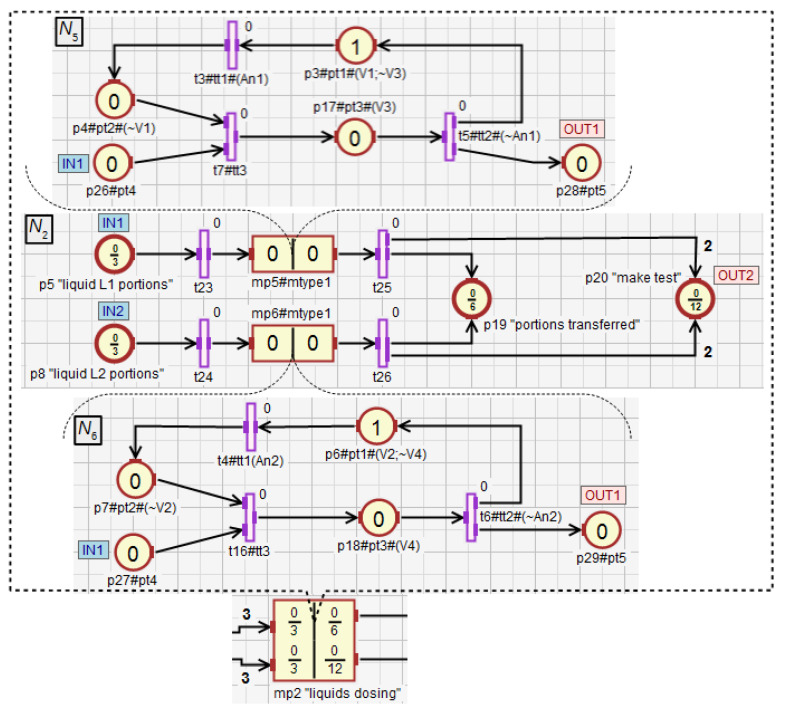
Dosing module for the liquids $L_{1}$ and $L_{2}$.

**Figure 15:**
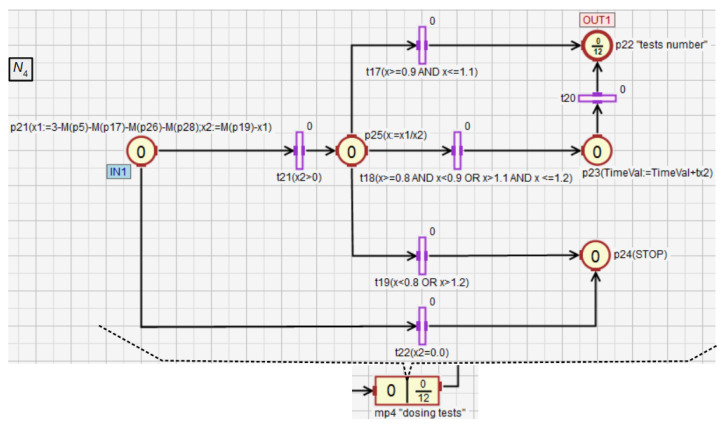
The module checking whether the liquids $L_{1}$ and $L_{2}$ are transferred to the mixer at a similar speed.

**Figure 16:**
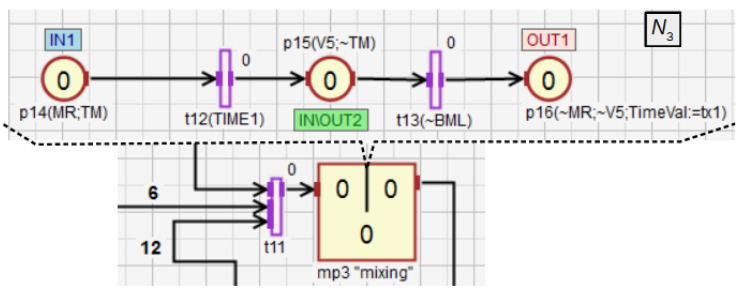
The module for mixing and emptying the mixer.

In this section, an example of using HFIPN is shown, including the concepts of macroplace instances and macroplaces that can have multiple input, output and input/output places. Figure 11 shows a system for mixing three components, while Figure 12, Figure 13, Figure 14, Figure 15 and Figure 16 illustrate an algorithm in the form of an HFIPN graph for controlling this system. First, three portions of each of the liquids $L_{1}$ and $L_{2}$, measured in the tanks ${\mathrm{Tk}}_{1}$ and ${\mathrm{Tk}}_{2}$, are transferred to the mixer. At the same time, two soluble bricks are fed into the mixer. Then all ingredients are mixed for some time represented by the variable TimeVal. The default time value is stored in ${\mathrm{tx}}_{1}$. Moreover, the measured time is converted to a value from the range [0, 1] and stored in TIME1. Finally, after obtaining the liquid of the desired consistency, the mixer is emptied and the whole process is repeated. In addition, if the liquids $L_{1}$ and $L_{2}$ are not transferred to the mixer at a similar speed, the mixing time can be extended even up to twelve times by the value of the variable ${\mathrm{tx}}_{2}$ or the entire process may stop.

Figure 12 shows the main subnet with four macroplaces (modules). The macroplace ${\mathrm{mp}}_{1}$ controls the transport of the bricks, while the macroplace ${\mathrm{mp}}_{2}$ controls the transfer of the liquids $L_{1}$ and $L_{2}$ to the mixer. The macroplace ${\mathrm{mp}}_{4}$ serves as a diagnostic module that checks the uniformity of filling by both liquids. The macroplace ${\mathrm{mp}}_{3}$ controls the mixing of all ingredients and emptying the mixer. The whole process starts with pressing the START button (transition $t_{1}$), and the red diode is turned on, while the green one is turned off (place $p_{2}$), what signals the operation of the system. The modules ${\mathrm{mp}}_{1}$ and ${\mathrm{mp}}_{2}$ are activated.

The macroplace ${\mathrm{mp}}_{1}$ (Figure 13) turns on the belt motor (place $p_{10}$), which causes that the two bricks are transported to the mixer. After the detection of the second brick by the binary transit detector TD the feed belt is turned off ($p_{13}$). The input place $p_{9}$ of type $p^{\prime \prime }$ represents the number of bricks that need to be transferred to the mixer. Moreover, this place enables to observe the state of the macroplace ${\mathrm{mp}}_{1}$ internal subnet ($N_{1}$) from the main subnet ($N_{0}$) without going inside the subnet. If the marking of this place equals 2, it means that the two bricks will soon be transported to the mixer. If it equals 1, a single brick has been transported and the other one will be moved soon. If the marking of this place is 0, and the marking of the output place $p_{13}$ equals 1, it means that there are two bricks in the mixer. In the other case, the brick transport module is not working.

In the macroplace ${\mathrm{mp}}_{2}$ (Figure 14), the transfer of the liquids $L_{1}$ and $L_{2}$ to the tanks ${\mathrm{Tk}}_{1}$ and ${\mathrm{Tk}}_{2}$ is controlled using macroplace ${\mathrm{mp}}_{5}$ and ${\mathrm{mp}}_{6}$, that are instances of the same type. The dosing of both liquids with the use of the valves $V_{1}$ (place $p_{3}$ in ${\mathrm{mp}}_{5}$) and $V_{2}$ ($p_{6}$ in ${\mathrm{mp}}_{6}$) is monitored by the analogue sensors ${\mathrm{An}}_{1}$ (transition $t_{3}$) and ${\mathrm{An}}_{2}$ ($t_{4}$). It ends when the value of the signal provided by the sensors equals 1. Then, both measured liquid portions are transferred from ${\mathrm{Tk}}_{1}$ and ${\mathrm{Tk}}_{2}$ to the mixer through the valves $V_{3}$ ($p_{17}$) and $V_{4}$ ($p_{18}$). The valve $V_{3}$ ($V_{4}$) is closed by the action assigned to the place $p_{3}$ ($p_{6}$) when the value from the liquid level sensor ${\mathrm{An}}_{1}$ (${\mathrm{An}}_{2}$) equals 0. Then, successive portions of liquids are measured.

The number of portions of the liquids $L_{1}$ and $L_{2}$ that need to be transferred to the mixer are represented by the input places $p_{5}$ and $p_{8}$. Both liquids have to be delivered at a similar speed due to the technological requirements. The sum of both portions of the liquids transferred into the mixer is represented by the output place $p_{19}$. Thus, at any time during the work of the liquid dosing module, its state can be determined based on the marking of the places $p_{5}$, $p_{8}$ and $p_{19}$ (analogously to ${\mathrm{mp}}_{1}$). If, for instance, the marking of the place $p_{5}$ equals 1.9, $p_{8}$ equals 1.87, and $p_{19}$ is 2, it means that the transfer of the first portions of both liquids is complete, while the second are being transferred into the dispensers. During the transport of the liquids to the mixer, twelve tests are performed to determine whether the speed of transferring the liquids $L_{1}$ and $L_{2}$ is similar, as long as the process has not been interrupted earlier. Through the output place $p_{20}$ the token is sent to the test module (the macroplace ${\mathrm{mp}}_{4}$). The test is performed every half of the portion obtained from both liquids (a total portion number is six).

The module comparing the speeds of the liquids dosing into the mixer (macroplace ${\mathrm{mp}}_{4}$) is shown in Figure 15. First, the numeric variables $x_{1}$ and $x_{2}$ are calculated (place $p_{21}$) based on the marking of places that are inside the macroplace ${\mathrm{mp}}_{2}$ to determine how many portions of $L_{1}$ and $L_{2}$ were transferred into the mixer. If $x_{2}$ equals 0 ($t_{22}$), the whole process is stopped ($p_{24}$). This most likely means the dispensing failure of $L_{2}$. If $x_{2}$ is greater than 0, the quotient of $x_{1}$ and $x_{2}$ is assigned to the variable x. Then, based on x, specific control actions are taken by activating one of the transitions $t_{17}$–$t_{19}$. Conditions excluding any conflict between these transitions are assigned to them. If $L_{1}$ and $L_{2}$ are transferred into the mixer evenly (x∈[0.9,1.1]), no additional control action is taken. If one of the liquids is transferred to the mixer too fast compared to the other, but their proportion is still sufficient (x∈[0.8,0.9)∪(1.1,1.2]), the mixing time of all components TimeVal is extended by ${\mathrm{tx}}_{2}$ ($p_{23}$). If $L_{1}$ and $L_{2}$ are transferred unevenly ($t_{19}$), the whole system is stopped ($p_{24}$).

In the macroplace ${\mathrm{mp}}_{3}$ (Figure 16), the mixing of all ingredients using the rotary engine MR is controlled. First, the operation of MR and the external timer TM are initialized ($p_{14}$). After mixing for a period of time defined by TimeVal and obtaining the liquid of the desired consistency, the mixer is emptied through the valve $V_{5}$ ($p_{15}$). The binary level sensor BML detects when the mixer is completely empty ($t_{13}$). After that, the mixing time TimeVal of the components is restored to the base value ($p_{16}$). Then, if the START button is on, the next cycle of the whole system starts. The place $p_{15}$ is an input-output place and allows for better visualization of the module from the main subnet.

The presented example shows that the use of HFIPN can improve the visualization of the process and facilitate communication between different modules in the net. Moreover, in the macroplace ${\mathrm{mp}}_{4}$ an example of dynamic control in response to fuzzy marking of places is presented.

## 5. Conclusions

In this work, a new concept of HFIPN has been introduced. The contributions of this study are as follows:

(a) Concept of a macroplace that can have several input, output and input-output places;
(b) Functionality of a macroplace instance;
(c) Formal definition of HFIPN;
(d) Concept and a definition of a hierarchy graph displaying a hierarchical structure and allowing a quick access to all subnets in an implementation version;
(e) Formal algebraic representation of HFIPN;
(f) Conversion of HFIPN to its flat version;
(g) Formal description of the combination of any two subnets in the hierarchical net.

The proposed macroplaces with several inputs and outputs can be useful for the implementation of modules that require asynchronous communication and facilitate resource modeling as illustrated in the example presented in Section 4. The concept of macroplace instances allows for the creation of several modules with the same structure and operation, their use in different areas of a net, and their simultaneous modification by introducing changes to one of them.

The nesting relations have also been defined, as well as the hierarchy graph which presents graphically the hierarchical structure of the net: the number of macroplaces and instances, and how they are nested in the net. The implementation of the graph in the HFIPN-SML simulator allows for quick movement between modules. An important part of the described research is an algebraic representation of HFIPN and the conversion method of a hierarchical net to the corresponding flat FIPN.

However, further studies on HFIPN are needed. In the future work, we are going to focus on the conversion of its graph to executable code.

## Figures and Tables

**Table 1:** Comparison of HFIPN to similar nets.

	HFIPN	GrafcetSFC	SIPN	Other Fuzzy PNs
hierarchy/modularization	**✓**	**✓**	**✓**	**✗**
analogue signals	**✓**	**✗**	**✗**	**✓**
software tool support	**✓**	**✓**	**✓**	**✗**
modeling of resources using a net structure	**✓**	**✗**	**✗**	**✗**
automatic executable code generation	**✓** /**✗**	**✓**	**✓**	**✗**
automatic investigation of properties	**✓** /**✗**	**✓** /**✗**	**✓**	**✗**

## Data Availability

Not applicable.

## References

[1] Valette R. (1979). Analysis of Petri nets by stepwise refinements. J. Comput. Syst. Sci..

[2] Suzuki I., Murata T. (1983). A method for stepwise refinement and abstraction of Petri nets. J. Comput. Syst. Sci..

[3] Vogler W., Tinhofer G., Schmidt G. (1986). Behaviour preserving refinements of Petri nets. Graph-Theoretic Concepts in Computer Science.

[4] Brauer W., Gold R., Vogler W., Rozenberg G. (1989). A survey of behaviour and equivalence preserving refinements of Petri nets. Advances in Petri Nets 1990.

[5] Bernardinello L., De Cindio F. (1992). A survey of basic net models and modular net classes. Advances in Petri Nets 1992.

[6] Van Der Aalst W.M. (2000). Workflow verification: Finding control-flow errors using Petri-net-based techniques. Business Process Management.

[7] Huang H., Jiao L., Cheung T.Y. (2012). Property-Preserving Petri Net Process Algebra in Software Engineering.

[8] Van Glabbeek R., Goltz U. (1989). Refinement of actions in causality based models. Stepwise Refinement of Distributed Systems Models, Formalisms, Correctness.

[9] Best E., Devillers R., Kiehn A., Pomello L. (1991). Concurrent bisimulations in Petri nets. Acta Inform..

[10] Vogler W. (1993). Bisimulation and action refinement. Theor. Comput. Sci..

[11] Van Glabbeek R., Goltz U. (2001). Refinement of actions and equivalence notions for concurrent systems. Acta Inform..

[12] Jiao L. (2008). Refining and verifying regular Petri nets. Int. J. Syst. Sci..

[13] Hack M. (1972). Analysis of Production Schemata by Petri Nets. Master’s Thesis.

[14] Lipton R.J. (1975). Reduction: A method of proving properties of parallel programs. Commun. ACM.

[15] Kwong Y.S. (1977). On reduction of asynchronous systems. Theor. Comput. Sci..

[16] Kowalk W., Valk R., Maurer H.A. (1979). On reductions of parallel programs. Automata, Languages and Programming.

[17] Berthelot G. (1985). Checking properties of nets using transformations. European Workshop on Applications and Theory in Petri Nets.

[18] Berthelot G., Brauer W., Reisig W., Rozenberg G. (1987). Transformations and decompositions of nets. Petri Nets: Central Models and Their Properties.

[19] Lee K.H., Favrel J. (1985). Hierarchical reduction method for analysis and decomposition of Petri nets. IEEE Trans. Syst. Man Cybern..

[20] Lee K.H., Favrel J., Baptiste P. (1987). Generalized Petri net reduction method. Syst. Man Cybern. IEEE Trans..

[21] Desel J. (1990). Reduction and design of well-behaved concurrent systems. CONCUR’90 Theories of Concurrency: Unification and Extension.

[22] Best E., Desel J. (1990). Partial order behaviour and structure of Petri nets. Form. Asp. Comput..

[23] Esparza J. (1994). Reduction and synthesis of live and bounded free choice Petri nets. Inf. Comput..

[24] Desel J., Esparza J. (2005). Free Choice Petri Nets.

[25] Jiao L., Cheung T.Y., Lu W. (2002). Characterizing liveness of Petri nets in terms of siphons. International Conference on Application and Theory of Petri Nets.

[26] Jiao L., Cheung T.Y., Lu W. (2004). On liveness and boundedness of asymmetric choice nets. Theor. Comput. Sci..

[27] Sloan R.H., Buy U. (1996). Reduction rules for time Petri nets. Acta Inform..

[28] Wang J., Deng Y., Zhou M. (2000). Compositional time Petri nets and reduction rules. IEEE Trans. Syst. Man Cybern. Part B.

[29] Juan E., Tsai J., Murata T., Zhou Y. (2001). Reduction methods for real-time systems using Delay Time Petri Nets. IEEE Trans. Softw. Eng..

[30] Fehling R., Rozenberg G. (1991). A concept of hierarchical Petri nets with building blocks. Advances in Petri Nets 1993.

[31] Jensen K. (1997). Coloured Petri Nets: Basic Concepts, Analysis Methods and Practical Use.

[32] Holvoet T., Verbaeten P. Petri charts: An alternative technique for hierarchical net construction. Proceedings of the 1995 IEEE International Conference on Systems, Man and Cybernetics, Intelligent Systems for the 21st Century.

[33] He X., Billington J., Reisig W. (1996). A formal definition of hierarchical predicate transition nets. Application and Theory of Petri Nets 1996.

[34] Andrzejewski G. (2005). Hierarchical Petri nets for digital controller design. Design of Embedded Control Systems.

[35] Pan H., Sun J. Complex knowledge system modeling based on hierarchical fuzzy petri net. Proceedings of the 2007 IEEE/WIC/ACM International Conferences on Web Intelligence and Intelligent Agent Technology-Workshops.

[36] (2013). International Standard IEC 60848:2013: Grafcet Specification Language for Sequential Function Charts Approach.

[37] (2013). International Standard IEC 61131-3: PROGRAMMABLE Controllers—Part 3: Programming Languages.

[38] David R., Alla H. (2010). Discrete, Continuous, and Hybrid Petri Nets.

[39] Silva M., Valette R. (1988). Petri nets and flexible manufacturing. European Workshop on Applications and Theory in Petri Nets.

[40] Valavanis K.P. (1990). On the hierarchical modeling analysis and simulation of flexible manufacturing systems with extended Petri nets. IEEE Trans. Syst. Man, Cybern..

[41] Zhou M., DiCesare F., Desrochers A.A. (1992). A hybrid methodology for synthesis of Petri net models for manufacturing systems. IEEE Trans. Robot. Autom..

[42] Jeng M.D., DiCesare F. (1995). Synthesis using resource control nets for modeling shared-resource systems. IEEE Trans. Robot. Autom..

[43] Zhou M. (1998). Modeling, analysis, simulation, scheduling, and control of semiconductor manufacturing systems: A Petri net approach. IEEE Trans. Semicond. Manuf..

[44] Ye J., Zhou M., Li Z., Al-Ahmari A. (2017). Structural decomposition and decentralized control of Petri nets. IEEE Trans. Syst. Man Cybern. Syst..

[45] Wiśniewski R., Karatkevich A., Wojnakowski M. (2019). Decomposition of distributed edge systems based on the Petri nets and linear algebra technique. J. Syst. Archit..

[46] An Y., Wu N., Zhao X., Li X., Chen P. (2018). Hierarchical colored petri nets for modeling and analysis of transit signal priority control systems. Appl. Sci..

[47] Sicchar J.R., Da Costa C.T., Silva J.R., Oliveira R.C., Oliveira W.D. (2018). A load-balance system design of microgrid cluster based on hierarchical petri nets. Energies.

[48] Gozhyj A., Kalinina I., Gozhyj V., Vysotska V. Web service interaction modeling with colored petri nets. Proceedings of the 2019 10th IEEE International Conference on Intelligent Data Acquisition and Advanced Computing Systems: Technology and Applications (IDAACS).

[49] Li W., He M., Sun Y., Cao Q. (2019). A novel layered fuzzy Petri nets modelling and reasoning method for process equipment failure risk assessment. J. Loss Prev. Process Ind..

[50] Yuan C., Liao Y., Kong L., Xiao H. (2021). Fault diagnosis method of distribution network based on time sequence hierarchical fuzzy petri nets. Electr. Power Syst. Res..

[51] Majma N., Babamir S.M., Monadjemi A. (2017). Runtime verification of pacemaker functionality using hierarchical fuzzy colored Petri-Nets. J. Med. Syst..

[52] Padberg J. (2018). Subtyping for hierarchical, reconfigurable Petri nets. arXiv.

[53] Figat M., Zieliński C. Methodology of designing multi-agent robot control systems utilising hierarchical petri nets. Proceedings of the 2019 International Conference on Robotics and Automation (ICRA).

[54] Silva J.M., Silva J.R. (2019). A new hierarchical approach to requirement analysis of problems in automated planning. Eng. Appl. Artif. Intell..

[55] Wisniewski R., Grobelna I., Karatkevich A. (2020). Determinism in cyber-physical systems specified by interpreted petri nets. Sensors.

[56] López J., Sánchez-Vilariño P., Sanz R., Paz E. (2020). Implementing autonomous driving behaviors using a message driven petri net framework. Sensors.

[57] Proth J.M., Xie X. (1996). Petri Nets: A Tool for Design and Management of Manufacturing Systems.

[58] Uzam M. (2004). The use of the Petri net reduction approach for an optimal deadlock prevention policy for flexible manufacturing systems. Int. J. Adv. Manuf. Technol..

[59] Gniewek L. (2013). Sequential control algorithm in the form of fuzzy interpreted Petri net. IEEE Trans. Syst. Man Cybern. Syst..

[60] Gniewek L. (2014). Coverability graph of fuzzy interpreted Petri net. IEEE Trans. Syst. Man Cybern. Syst..

[61] Markiewicz M., Surdej Ł., Gniewek L. Transformation of a fuzzy interpreted Petri net diagram into structured text code. Proceedings of the 2016 21st International Conference on Methods and Models in Automation and Robotics (MMAR).

[62] Markiewicz M., Gniewek L. (2017). A program model of fuzzy interpreted Petri net to control discrete event systems. Appl. Sci..

[63] Hajduk Z., Wojtowicz J. (2020). FPGA implementation of fuzzy interpreted Petri net. IEEE Access.

[64] Markiewicz M., Gniewek L. (2017). Conception of hierarchical fuzzy interpreted Petri net. Stud. Inf. Control.

[65] Frey G. Automatic implementation of Petri net based control algorithms on PLC. Proceedings of the 2000 American Control Conference, ACC (IEEE Cat. No. 00CH36334).

[66] Minas M., Frey G. Visual PLC-programming using signal interpreted Petri nets. Proceedings of the 2002 American Control Conference.

[67] Klein S., Frey G., Minas M. (2003). PLC programming with signal interpreted Petri nets. International Conference on Application and Theory of Petri Nets.

[68] Frey G. (2003). Hierarchical design of logic controllers using signal interpreted Petri nets. IFAC Proc. Vol..

[69] Andreu D., Pascal J.C., Valette R. (1997). Fuzzy Petri net-based programmable logic controller. IEEE Trans. Syst. Man Cybern. Part B.

[70] Gniewek L., Kluska J. (2004). Hardware implementation of fuzzy Petri net as a controller. IEEE Trans. Syst. Man Cybern. Part B.

[71] Venkateswaran P., Bhat J. (2006). Fuzzy Petri net algorithm for flexible manufacturing systems. ACSE J..

[72] Bauer N., Engell S., Huuck R., Lohmann S., Lukoschus B., Remelhe M., Stursberg O. (2004). Verification of PLC programs given as sequential function charts. Integration of Software Specification Techniques for Applications in Engineering.

[73] Roussel J.M., Lesage J.J. Validation and verification of Grafcets using state machine. IMACS-IEEE “CESA’96” July, Lille, 1996.

[74] Provost J., Roussel J.M., Faure J.M. A formal semantics for Grafcet specifications. Proceedings of the 2011 IEEE International Conference on Automation Science and Engineering.

[75] Remelhe M., Lohmann S., Stursberg O., Engell S., Bauer N. Algorithmic verification of logic controllers given as sequential function charts. Proceedings of the 2004 IEEE International Conference on Robotics and Automation (IEEE Cat. No. 04CH37508).

[76] Lohmann S., Stursberg O., Engell S. Comparison of event-triggered and cycle-driven models for verifying SFC programs. Proceedings of the 2007 American Control Conference.

[77] L’Her D., Le Parc P., Marcé L. (2001). Proving sequential function chart programs using timed automata. Theor. Comput. Sci..

[78] Stursberg O., Lohmann S. Analysis of logic controllers by transformation of SFC into timed automata. Proceedings of the 44th IEEE Conference on Decision and Control.

[79] Wightkin N., Buy U., Darabi H. (2010). Formal modeling of sequential function charts with time Petri nets. IEEE Trans. Control Syst. Technol..

[80] Sogbohossou M., Vianou A. (2015). Formal modeling of Grafcets with time Petri nets. IEEE Trans. Control Syst. Technol..

[81] Peng S., Zhou M. Design and analysis of sequential function charts using sensor-based stage Petri nets. Proceedings of the SMC’03 Conference Proceedings, 2003 IEEE International Conference on Systems, Man and Cybernetics, Conference Theme-System Security and Assurance (Cat. No. 03CH37483).

[82] Fujino K., Imafuku K., Yuh Y., Hirokazu N. (2000). Design and verification of the SFC program for sequential control. Comput. Chem. Eng..

[83] Schumacher F., Fay A. Transforming time constraints of a Grafcet graph into a suitable Petri net formalism. Proceedings of the 2013 IEEE International Conference on Industrial Technology (ICIT).

